# Supplementary material for: Structure of the Sec14 domain of Kalirin reveals a distinct class of lipid-binding module in RhoGEFs
Source: Nat Commun. 2023 Jan 6;14:96. doi: 10.1038/s41467-022-35678-4 (PMC9823006; doi:10.1038/s41467-022-35678-4)
Supplement: Supplementary file 1 — Supplementary Information [file 41467_2022_35678_MOESM1_ESM.pdf]

Supplementary Information

Structure of the Sec14 domain of Kalirin reveals a new class of lipid-binding module in RhoGEFs

Yunfeng Li[1], Yulia Pustovalova[1], Tzanko I. Doukov[2], Jeffrey C. Hoch[1], Richard E. Mains[3], Betty A. Eipper[1,3] and Bing Hao[1, *]

[1]Department of Molecular Biology and Biophysics, University of Connecticut Health Center, Farmington, Connecticut 06030, USA;

[2]Macromolecular Crystallography Group, Stanford Synchrotron Radiation Light Source, SLAC National Accelerator Laboratory, Stanford University, Stanford, California 94309, USA;

[3]Department of Neuroscience, University of Connecticut Health Center, Farmington, Connecticut 06030, USA.

[*]Corresponding author: Bing Hao (bhao@uchc.edu)

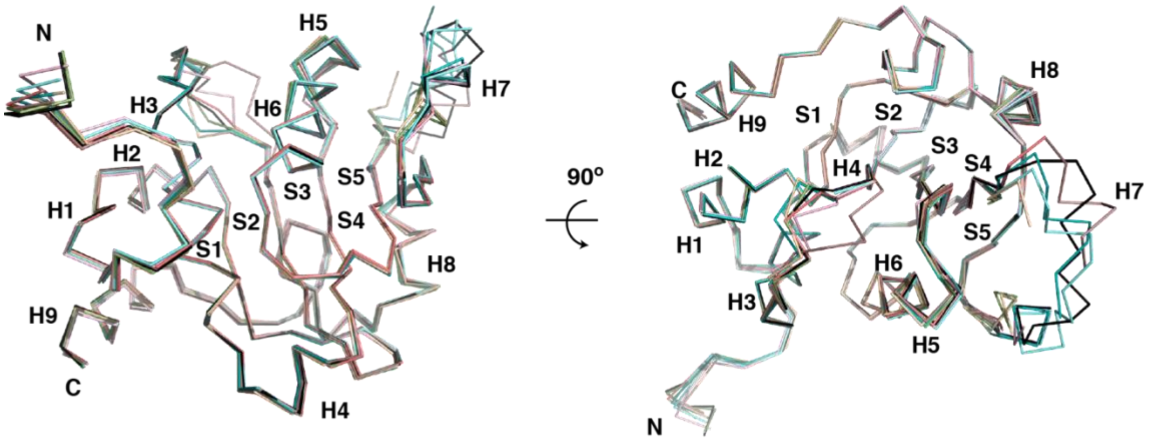

Supplementary Figure 1. Superposition of the eight Kal^{bSec14} molecules in the crystallographic asymmetric unit.

The structures of the monomers can be superimposed with an overall rmsd of 0.8 Å over 175 C_α atoms. The regions where the structures diverge are in the N terminus, the S2–H3 loop and the H7–S5 loop; in four of the molecules, the H7–S5 loop lacks interpretable electron densities and is presumably disordered. The conformational plasticity of these regions is consistent with their dynamic feature observed in NMR spectra in solution (see Fig. 3).

a

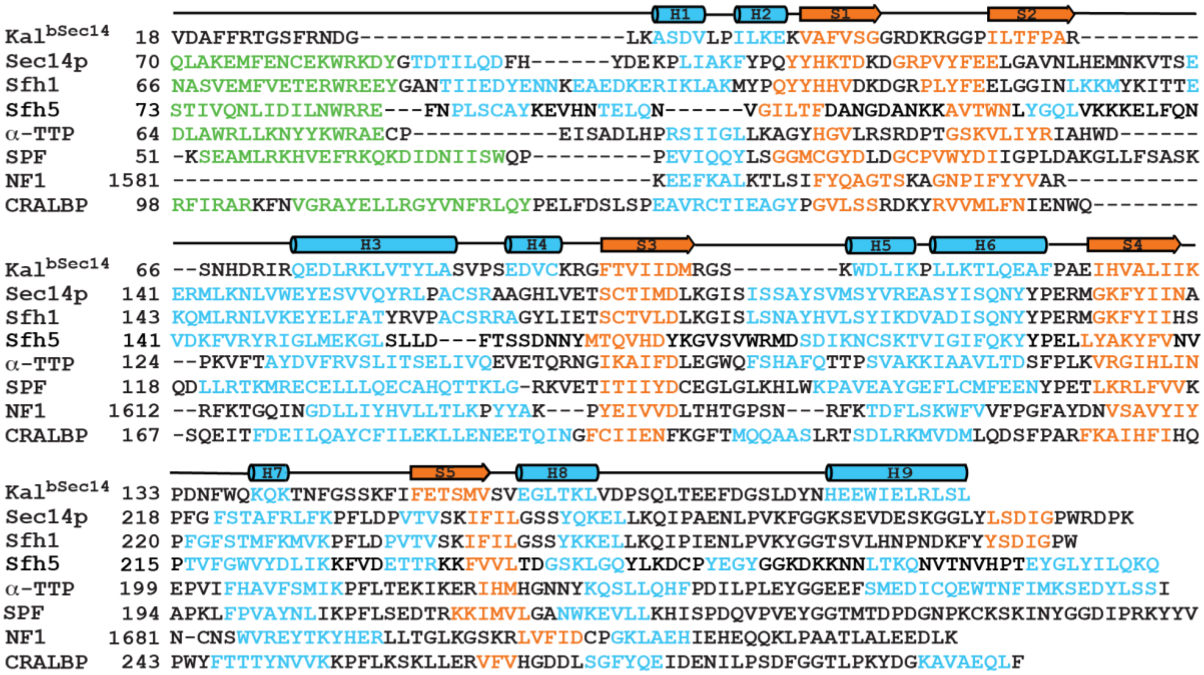

b

Name	PDB ID	Ligand	Sequence Identity to Kal^{bSec14} (%)	RMSD of C_α atoms to Kal^{bSec14} (Å)
Sec14p	1AUA	βOG	16.1	4.0
Sfh1	3B7N	PtdIns	13.2	3.3
Sfh5	6W32	Heme	11.5	3.8
α-TTP	1OIZ	α-Toc	12.8	4.2
SPF	1O6U	β-octylglucoside	14.3	4.9
NF1	2D4Q	Triton X-100	13.7	3.3
CRALBP	3HY5	Retinal	17.1	4.4

c

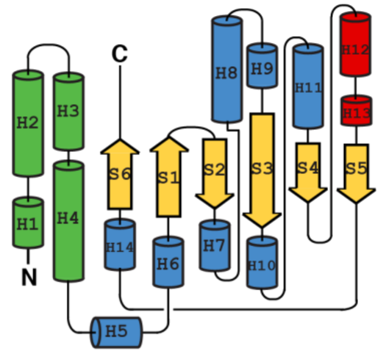

Supplementary Figure 2. Sequence and structure-based alignment of Kal^{bSec14} and select Sec14 superfamily proteins.

a. Structure-based sequence alignment of Kal^{bSec14} and Sec14 superfamily members with known structures, including Sec14p (PDB ID code 1AUA), Sfh1 (3B7N), Sfh5 (6W32), α-TTP (1OIZ), SPF (1O6U), NF1 (2D4Q) and CRALBP (3HY5). Each Sec14 protein structure was superimposed with that of Kal^{bSec14} and the overlaid secondary-structure elements were used as the guide for the alignment. Secondary-structure elements for C_α atoms are shown as blue cylinders (helices) and orange arrows (β strands). Residues found in helices and β strands are colored in blue and orange, respectively; the helices belonging to the CRAL_TRIO_N domain, which is absent from Kal^{bSec14}, are shown in green.
b. Sequence identity and overall rmsd of C_α atoms of the Sec14 family proteins in panel a to Kal^{bSec14}. The bound ligand or detergent in each crystal structure is also listed.
c. Topology diagram of yeast prototype Sec14p (PDB ID code 1AUA). Cylinders and arrows represent helices and β strands, respectively. The CRAL_TRIO_N domain is shown in green.

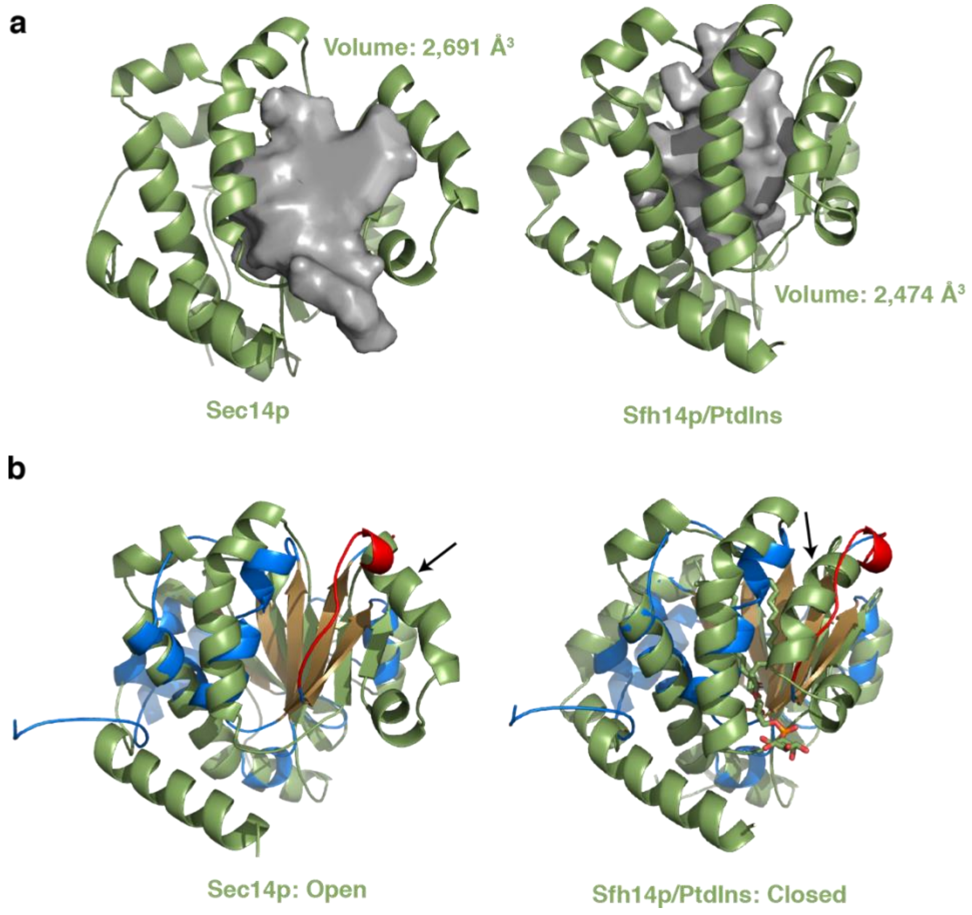

Supplementary Figure 3. Structural comparison of Kal^{bSec14} and select Sec14 family proteins.

a. Structures of apo-Sec14p (PDB ID code 1AUA; left) and Sfh1 bound to phosphatidylinositol (PtdIns; PDB ID code 3B7N; right) are shown; the CRAL_TRIO_N domain in both structures, and the ligand in the Sfh1 structure were omitted for clarity. Representations of the internal pockets were generated by HOLLOW [1]. Pocket volumes were calculated with CASTp [2].

b. Superposition of structure of Kal^{bSec14} with apo-Sec14p in its open conformation (green; left) and Sfh1/PtdIns in its closed conformation (green; right), respectively. The PtdIns molecule is shown as licorice sticks. The gating helices in Sec14p and Sfh1 are indicated with a black arrow.

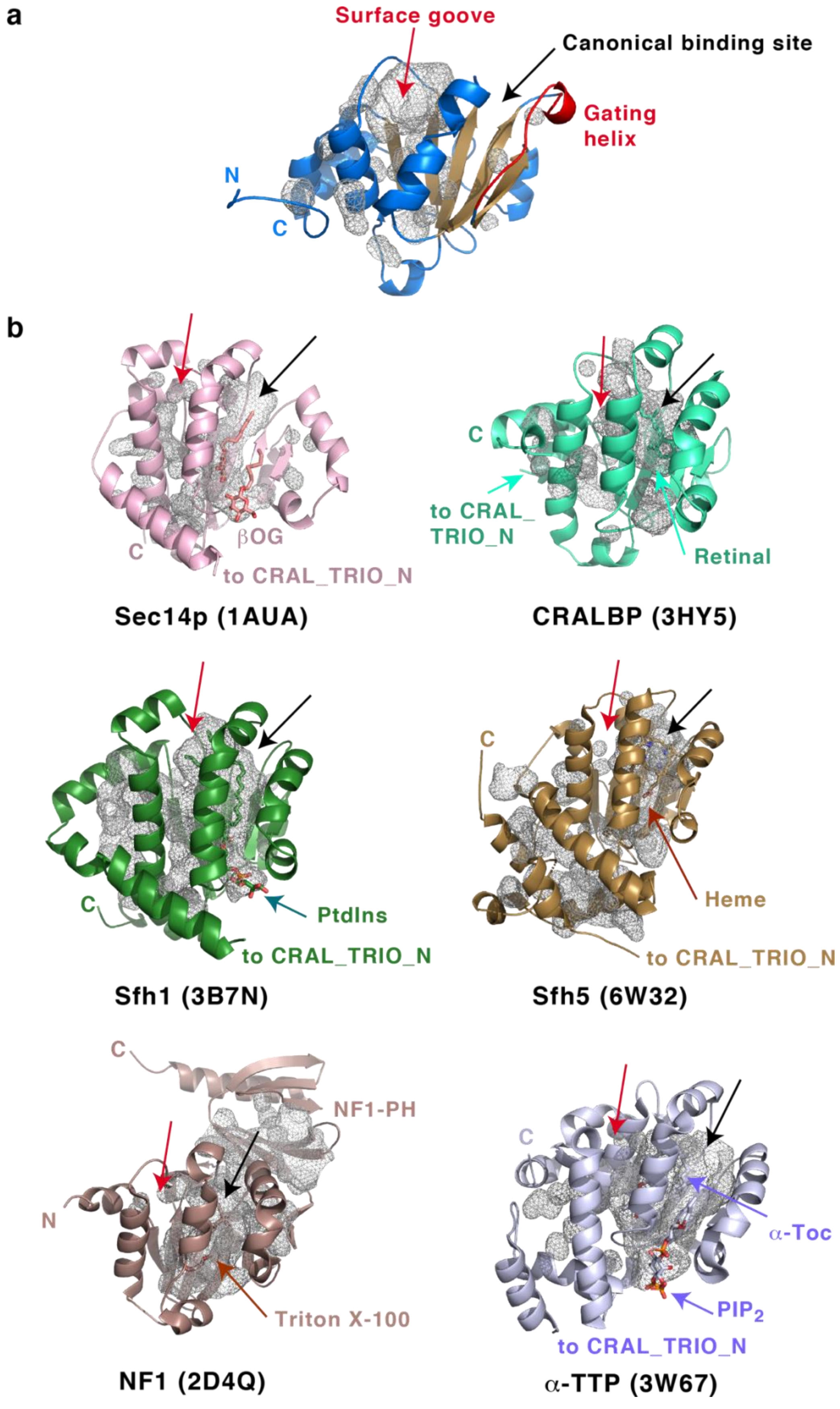
a
Surface goove
Canonical binding site
Gating helix
N
C
b
βOG
C
to CRAL_TRIO_N
Sec14p (1AUA)
C
to CRAL_TRIO_N
Retinal
CRALBP (3HY5)
C
PtdIns
to CRAL_TRIO_N
Sfh1 (3B7N)
C
Heme
to CRAL_TRIO_N
Sfh5 (6W32)
C
NF1-PH
N
Triton X-100
NF1 (2D4Q)
C
α-Toc
PIP2
to CRAL_TRIO_N
α-TTP (3W67)

Supplementary Figure 4. Pockets in the structures of Kal^{bSec14} and select Sec14 family proteins.

a. Surface representation of the inner and surface cavities of Kal^{bSec14}. The cavity detection radius and cutoff are set at 5 Å and 3 Å, respectively, as defined by Pymol. The locations for the surface groove and the presumed canonical ligand binding site are indicated by red and black arrows, respectively.

b. Structures of Sec14p[3] (PDB ID code 1AUA), CRALBP[4] (3HY5), Sfh1[5] (3B7N), Sfh5[6] (6W32), NF1[7] (2D4Q) and α-TTP[8] (3W67) in an orientation similar to that of Kal^{bSec14} shown in panel a. Surface representation of the inner and surface pockets are shown. The bound ligands in the structures are shown as licorice sticks. The location for the presumed surface groove and the canonical ligand binding site are indicated by red and black arrows, respectively.

a

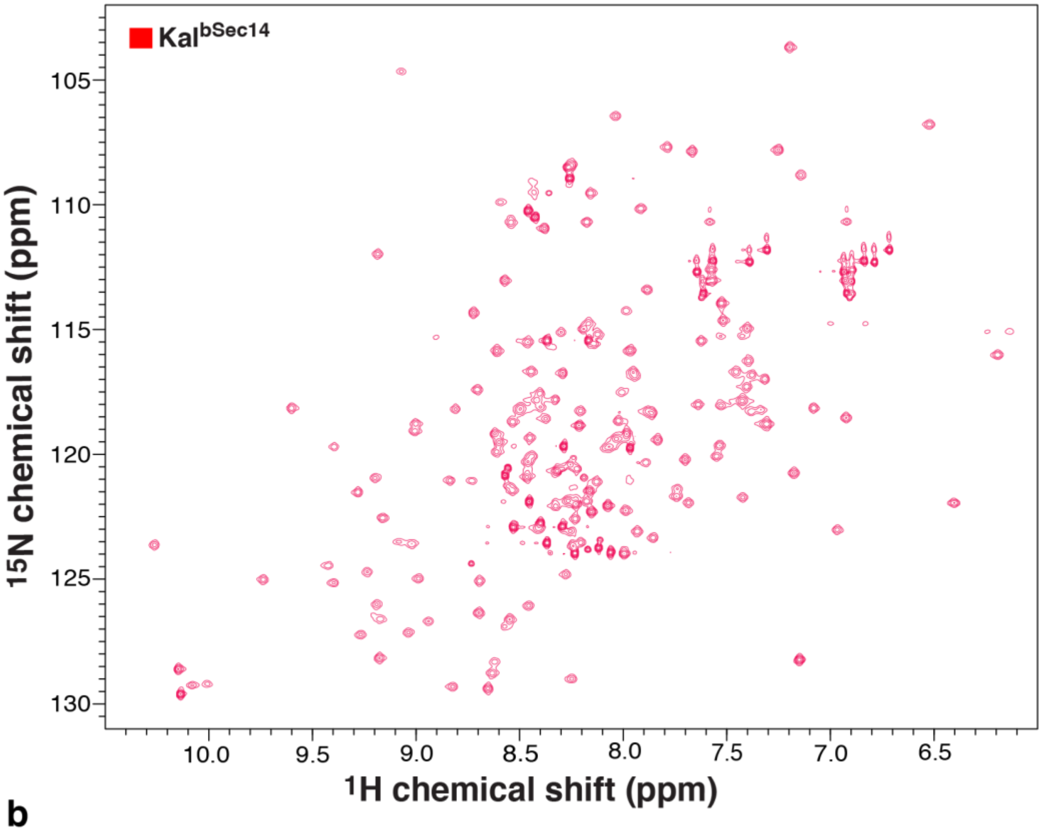

Kal[bSec14]
15N chemical shift (ppm)
105
110
115
120
125
130
10.0
9.5
9.0
8.5
8.0
7.5
7.0
6.5
1H chemical shift (ppm)

b

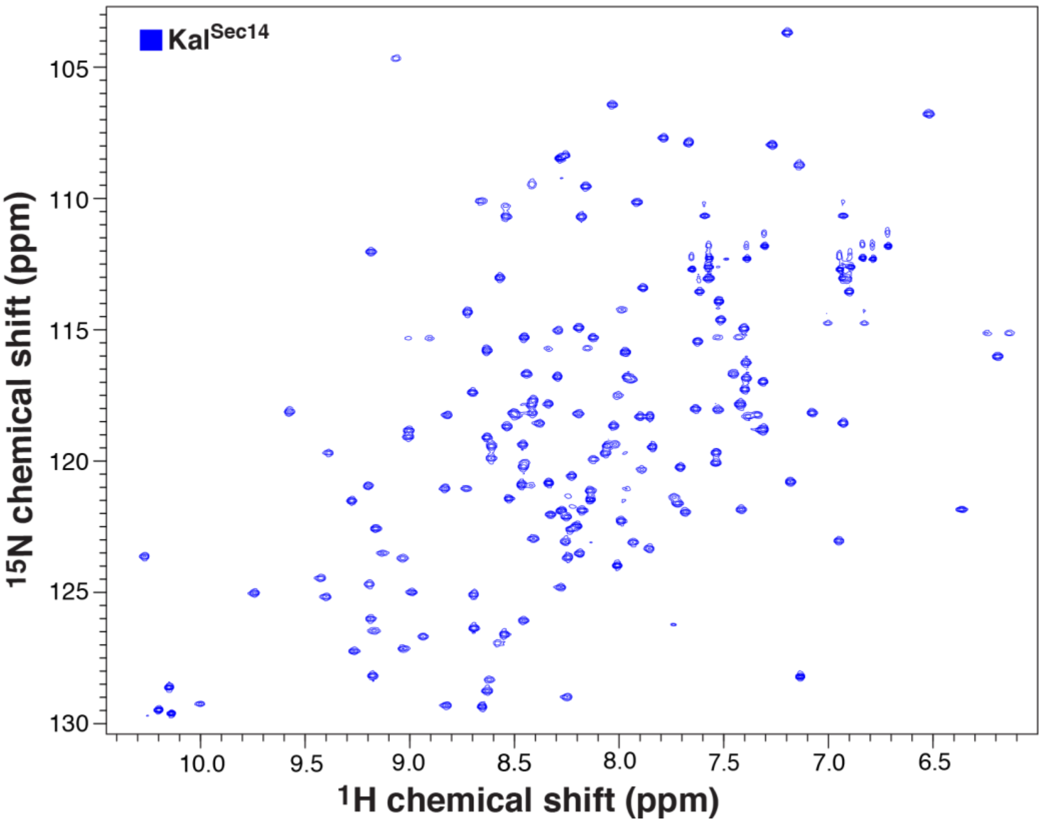

Kal[Sec14]
15N chemical shift (ppm)
105
110
115
120
125
130
10.0
9.5
9.0
8.5
8.0
7.5
7.0
6.5
1H chemical shift (ppm)

c

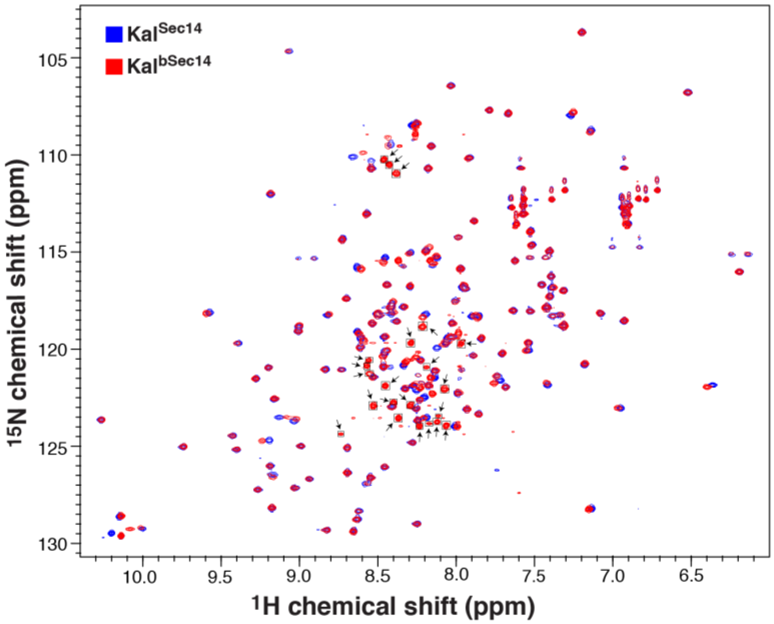

KalSec14
KalbSec14
15N chemical shift (ppm)
1H chemical shift (ppm)
105
110
115
120
125
130
10.0
9.5
9.0
8.5
8.0
7.5
7.0
6.5

d

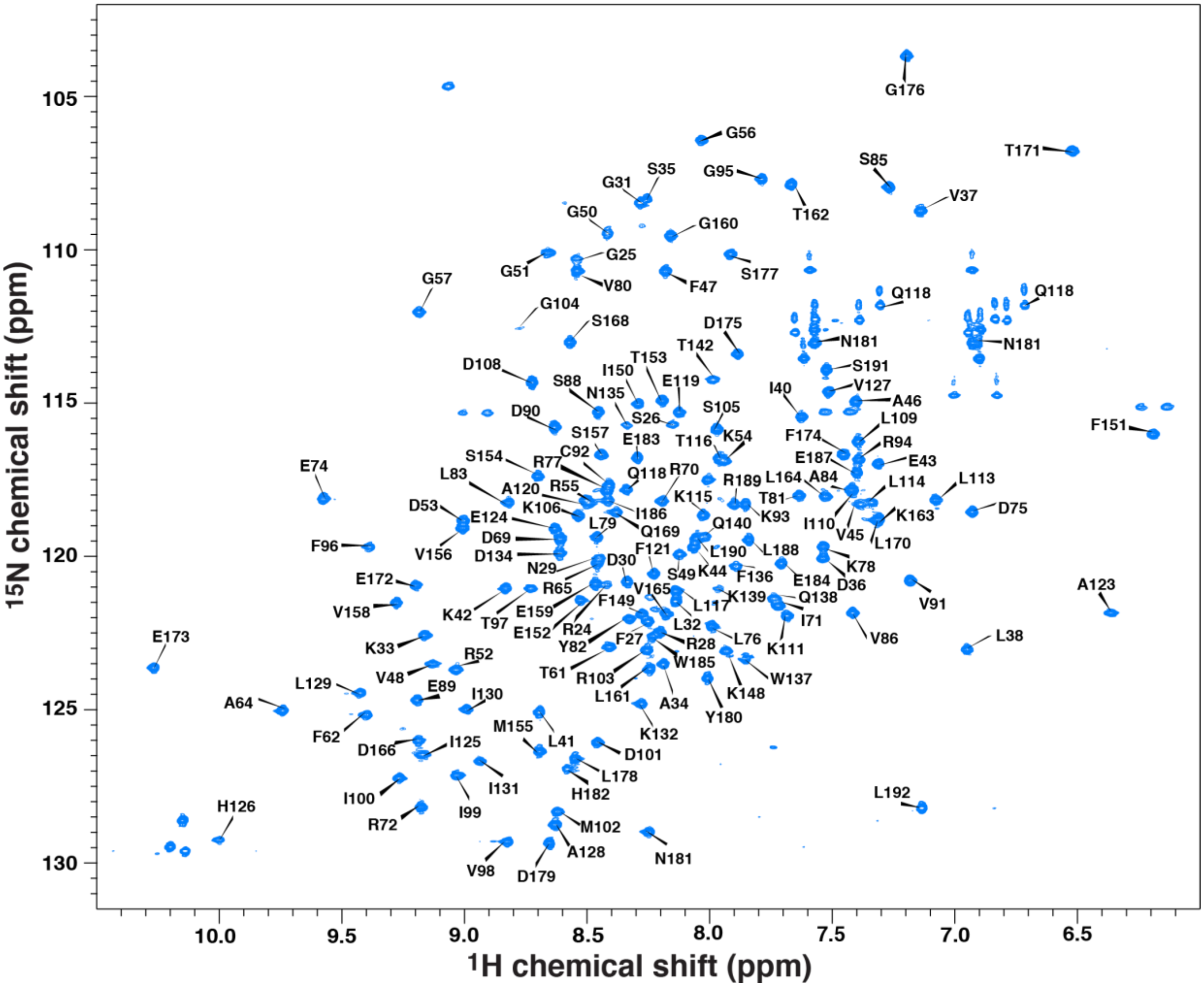

15N chemical shift (ppm)
1H chemical shift (ppm)
105
110
115
120
125
130
10.0
9.5
9.0
8.5
8.0
7.5
7.0
6.5
G176
G56
T171
S85
G95
S35
G31
V37
T162
G50
G160
G25
G51
S177
G57
V80
F47
Q118
Q118
G104
S168
D175
N181
N181
T142
S191
D108
T153
I150
E119
V127
S88
N135
I40
A46
D90
S26
S105
L109
S157
T116
K54
F174
F151
E183
R94
S154
R77
C92
E187
E43
E74
L83
Q118
R70
L164
A84
L113
A120
R55
R189
L114
D53
K106
K115
T81
I186
D75
K93
E124
L79
I110
K163
Q169
Q140
V45
D69
L170
F96
F121
L190
L188
V156
D134
K78
D30
K44
E184
N29
S49
F136
D36
E172
A123
R65
V165
K139
V91
Q138
E159
L117
K42
F149
I71
V158
T97
L32
V86
R24
E152
L76
E173
F27
K111
L38
Y82
R28
K33
R52
W185
W137
T61
V48
R103
K148
L129
E89
L161
A34
I130
Y180
A64
K132
F62
M155
L41
I125
D166
D101
L178
I131
H182
I100
L192
I99
H126
R72
M102
A128
N181
V98
D179

Supplementary Figure 5. 2D ^{1}H-^{15}N HSQC spectra of Kal^{bSec14} and Kal^{Sec14} with sequence-specific assignments.

a. ^{1}H-^{15}N HSQC spectrum of ^{15}N-labeled apo Kal^{bSec14}.

b. ^{1}H-^{15}N HSQC spectrum of ^{15}N-labeled apo Kal^{Sec14}.

c. Overlay of ^{1}H-^{15}N HSQC spectra of Kal^{Sec14} (blue) and Kal^{bSec14} (red). The 22 unique peaks present in the spectrum of Kal^{bSec14} but not in that of Kal^{Sec14} are indicated by a square and an arrow. The majority of these peaks are clustered in the middle part of the spectrum corresponding to the N-terminal disordered region, while the three presumed glycine peaks are found on the upper left part of the spectrum.

d. Fully annotated ^{1}H-^{15}N HSQC spectrum of Kal^{Sec14}. Assigned peaks are labeled with the single letter code of the corresponding residue followed by the number in the protein sequence.

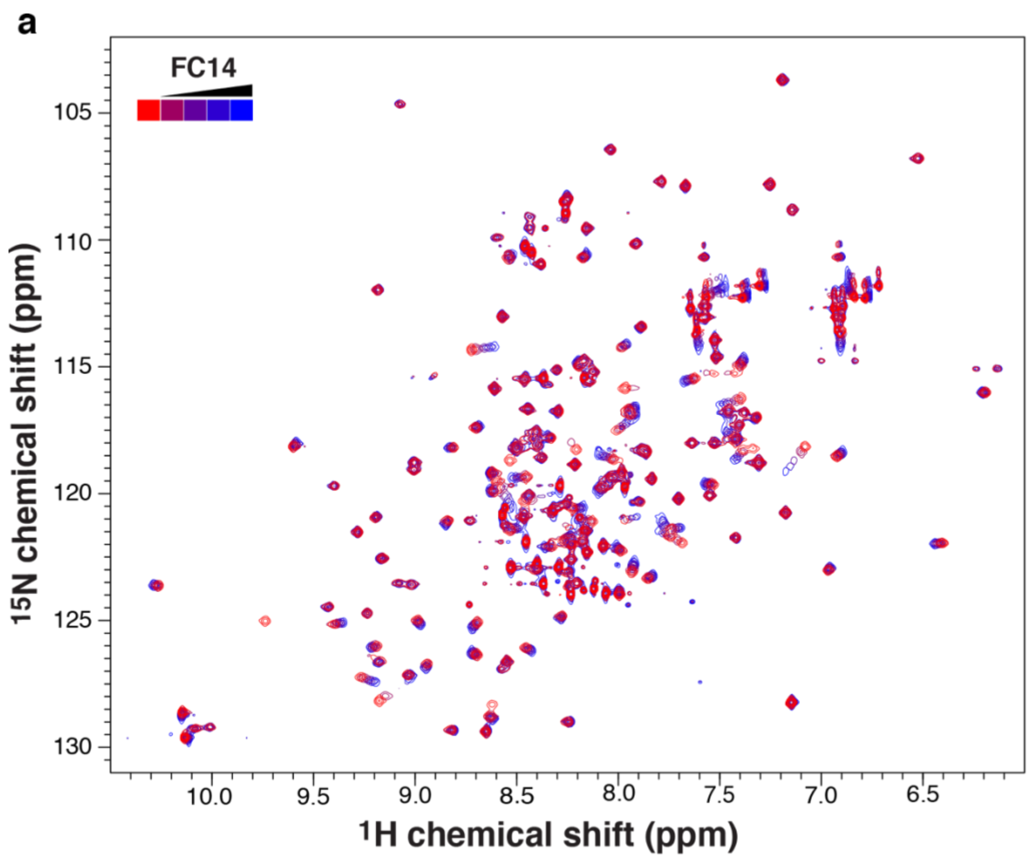
a
FC14
105
110
115
120
125
130
15N chemical shift (ppm)
10.0
9.5
9.0
8.5
8.0
7.5
7.0
6.5
1H chemical shift (ppm)

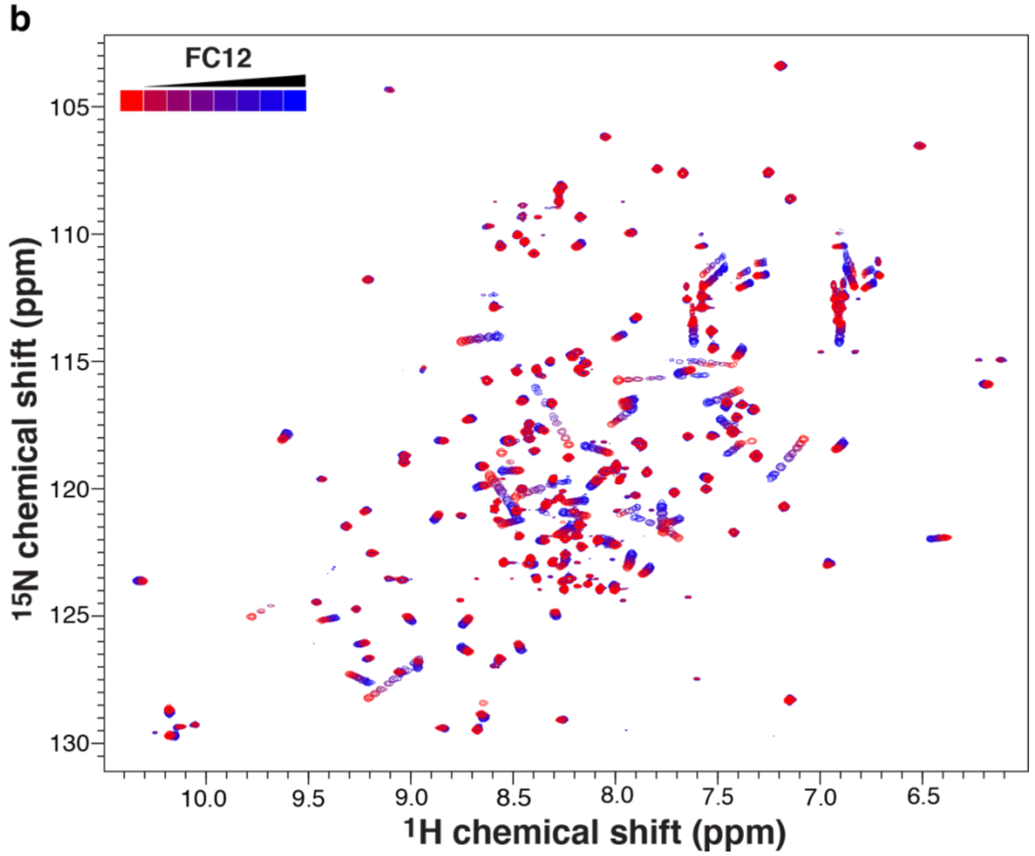
b
FC12
105
110
115
120
125
130
15N chemical shift (ppm)
10.0
9.5
9.0
8.5
8.0
7.5
7.0
6.5
1H chemical shift (ppm)

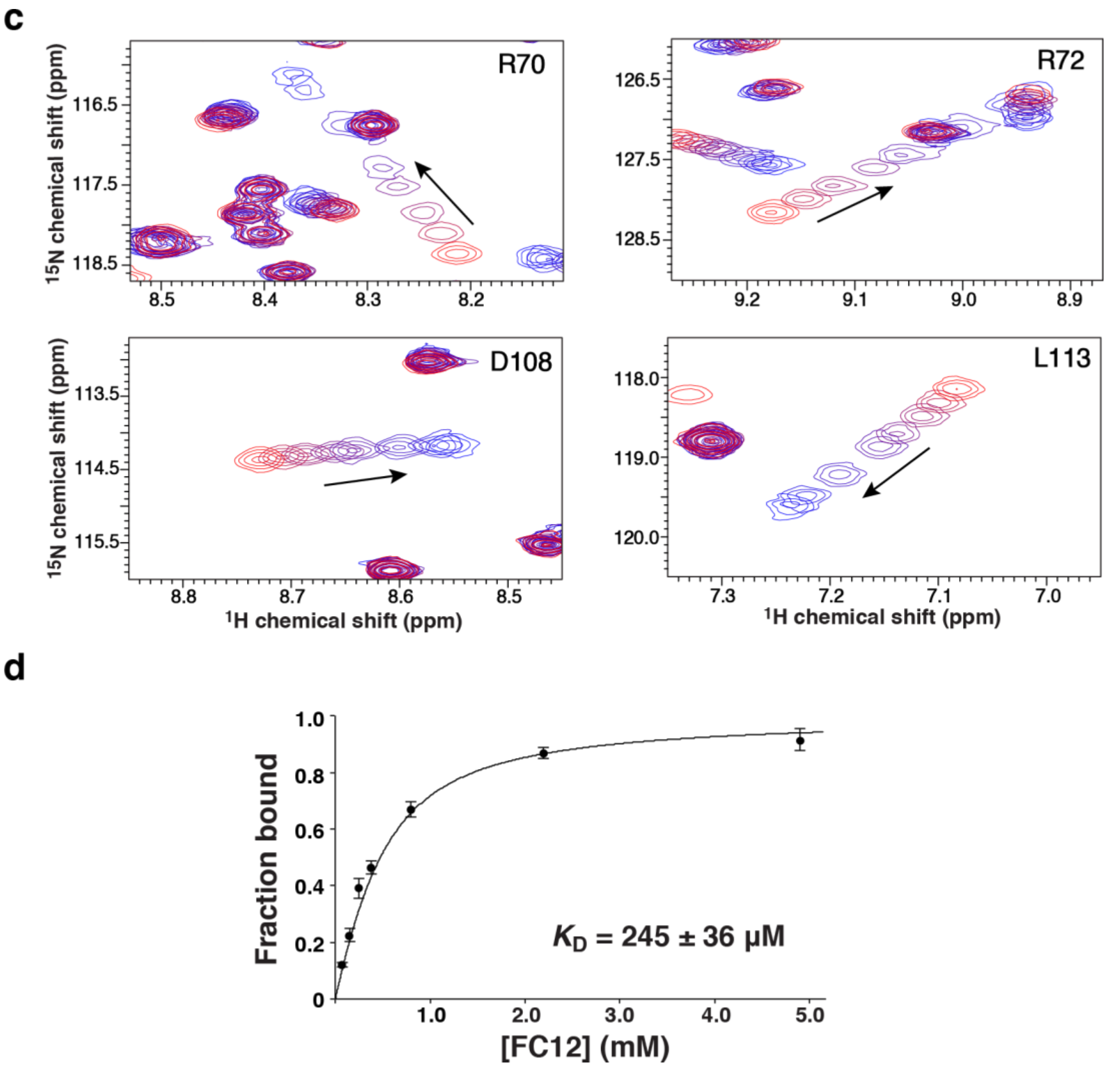

Supplementary Figure 6. Kal^{bSec14} binds PC detergents FC14 and FC12.

a, b. Series of ^{1}H-^{15}N HSQC spectra recorded during titration of Kal^{bSec14} with increasing amounts of FC14 (a) or FC12 (b).

c. Four residues of Kal^{bSec14} that demonstrate CSPs when titrated with increasing amounts of FC12 (1:25 final molar ratio). For each residue, the cross peaks are color-ramped from red to blue with increasing FC12 concentrations, as indicated by arrow.

d. A plot of normalized global fitting of the averaged CSPs ($\Delta\delta_{obs}/\Delta\delta_{max}$) as a function of FC12 concentration to estimate the K_D for binding. The fitting data and the K_D value represent the mean ± S.D. of the CSP data for individual residues (n = 11). Source data are provided as a Source Data file.

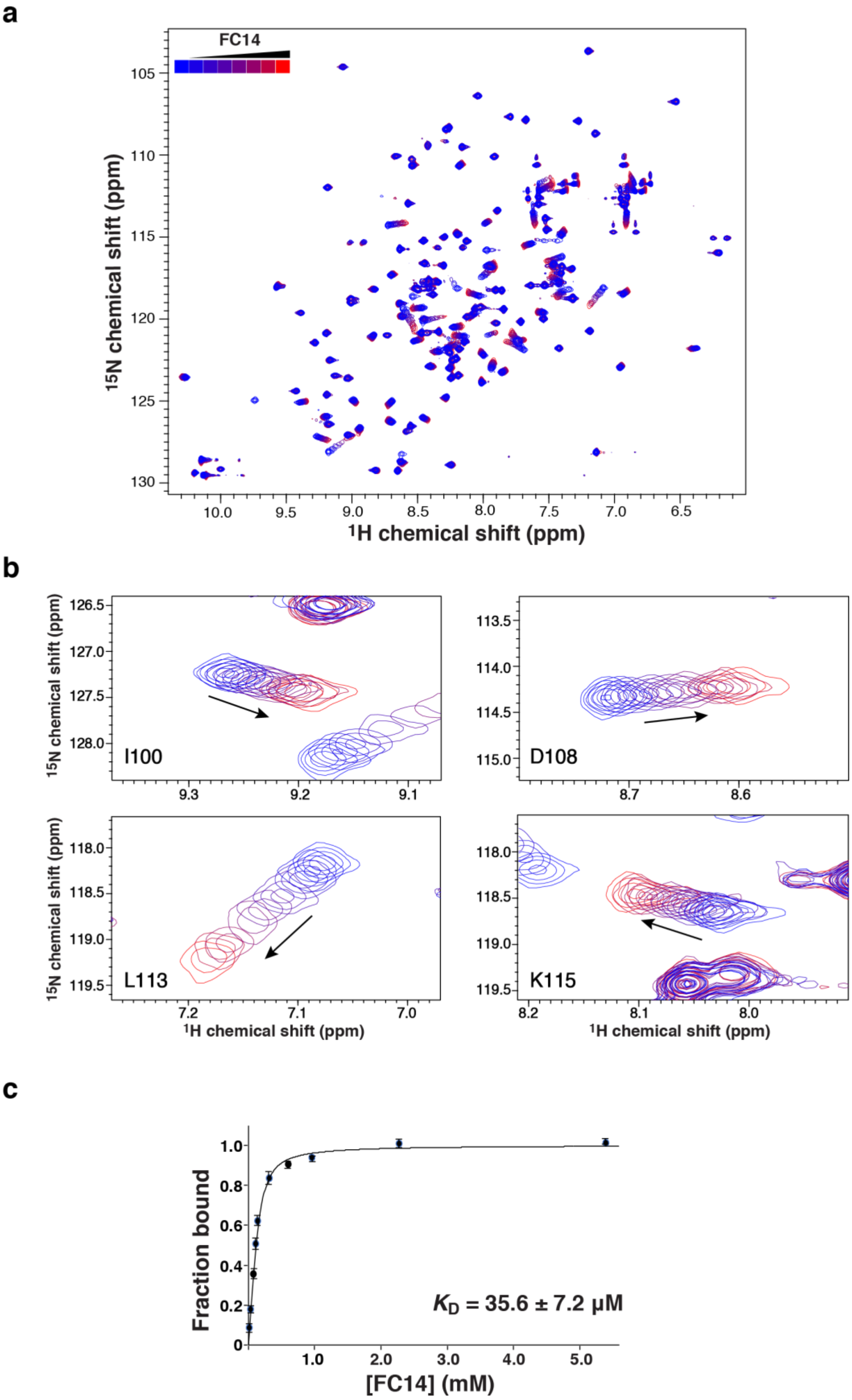
a
FC14
15N chemical shift (ppm)
1H chemical shift (ppm)
b
I100
D108
L113
K115
15N chemical shift (ppm)
1H chemical shift (ppm)
c
Fraction bound
[FC14] (mM)
KD = 35.6 ± 7.2 μM

d

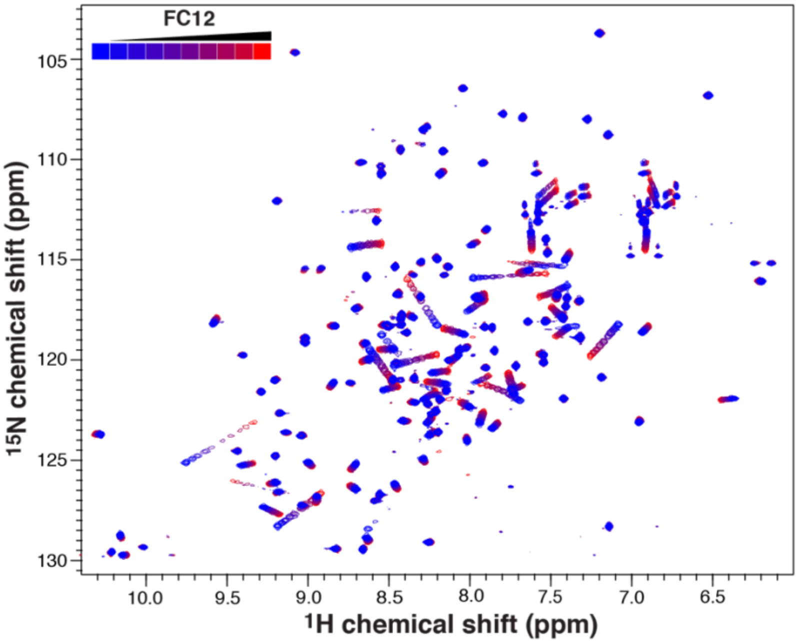
FC12
105
110
115
120
125
130
^{15}N chemical shift (ppm)
10.0
9.5
9.0
8.5
8.0
7.5
7.0
6.5
^{1}H chemical shift (ppm)

e

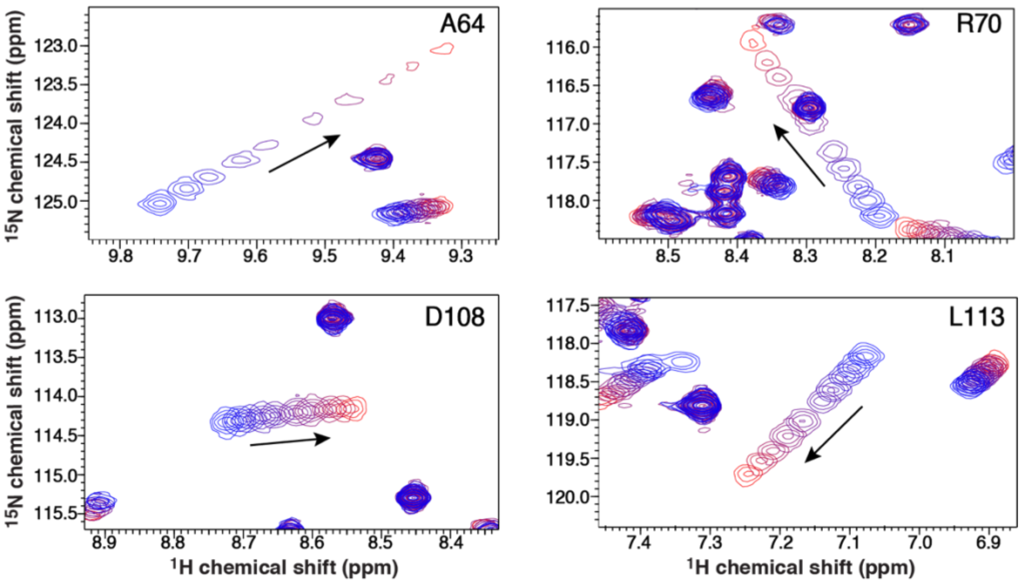
A64
R70
D108
L113
^{15}N chemical shift (ppm)
^{1}H chemical shift (ppm)

f

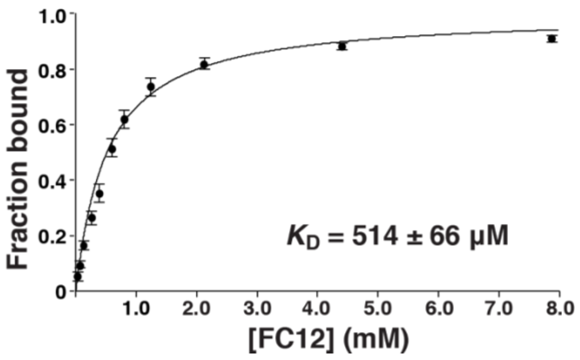
Fraction bound
K_D = 514 ± 66 μM
[FC12] (mM)

Supplementary Figure 7. Kal^{Sec14} binds PC detergents FC14 and FC12.

a, d. Series of ^{1}H-^{15}N HSQC spectra recorded during titration of Kal^{Sec14} with increasing amounts of FC14 (a) or FC12 (d).

b, e. Four residues of Kal^{Sec14} that demonstrate CSPs when titrated with increasing amounts of FC14 (b; 1:10 final molar ratio) or FC12 (e; 1:20 final molar ratio). For each residue, the cross peaks are color-ramped from blue to red with increasing FC14 or FC12 concentrations, as indicated by arrow.

c, f. Plots of normalized global fitting of the averaged CSPs ($\Delta\delta_{obs}/\Delta\delta_{max}$) as a function of FC14 (c) or FC12 (f) concentration to estimate the K_D for binding. The fitting data and the K_D values represent the mean ± S.D. of the CSP data fitting for individual residues (n = 24 for FC14; n = 20 for FC12). Source data are provided as a Source Data file.

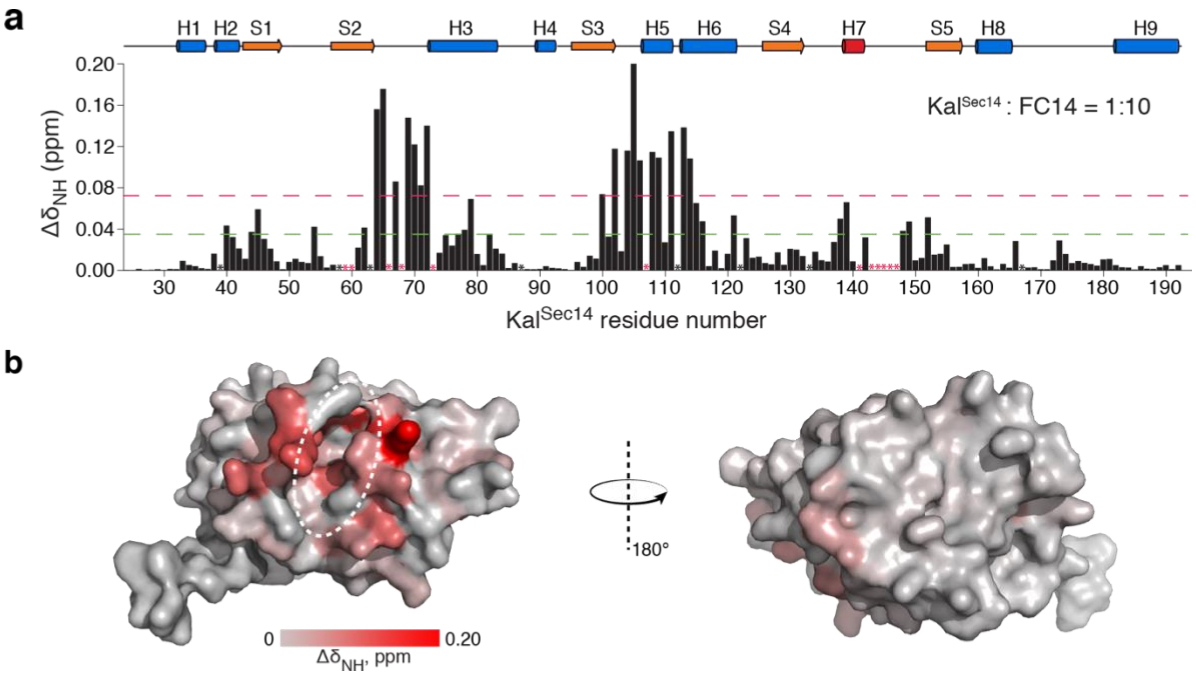

Supplementary Figure 8. Kal^{Sec14} binds FC14 via the surface groove.

a. Plot of per-residue backbone CSPs between free and FC14-bound states of Kal^{Sec14}. Proline residues and residues missing backbone assignment are indicated by asterisks (black, proline; red, unassigned). Dashed green and red lines indicate CSP values within one (1σ) and two (2σ) S.D. of the average CSP (0.037 ppm) among all assigned residues, respectively. Source data are provided as a Source Data file.

b. Surface representation of Kal^{Sec14} colored according to CSPs induced by FC14 binding, from light gray (no observed CSP) to red (maximum CSP). Dashed ellipse indicates the surface groove.

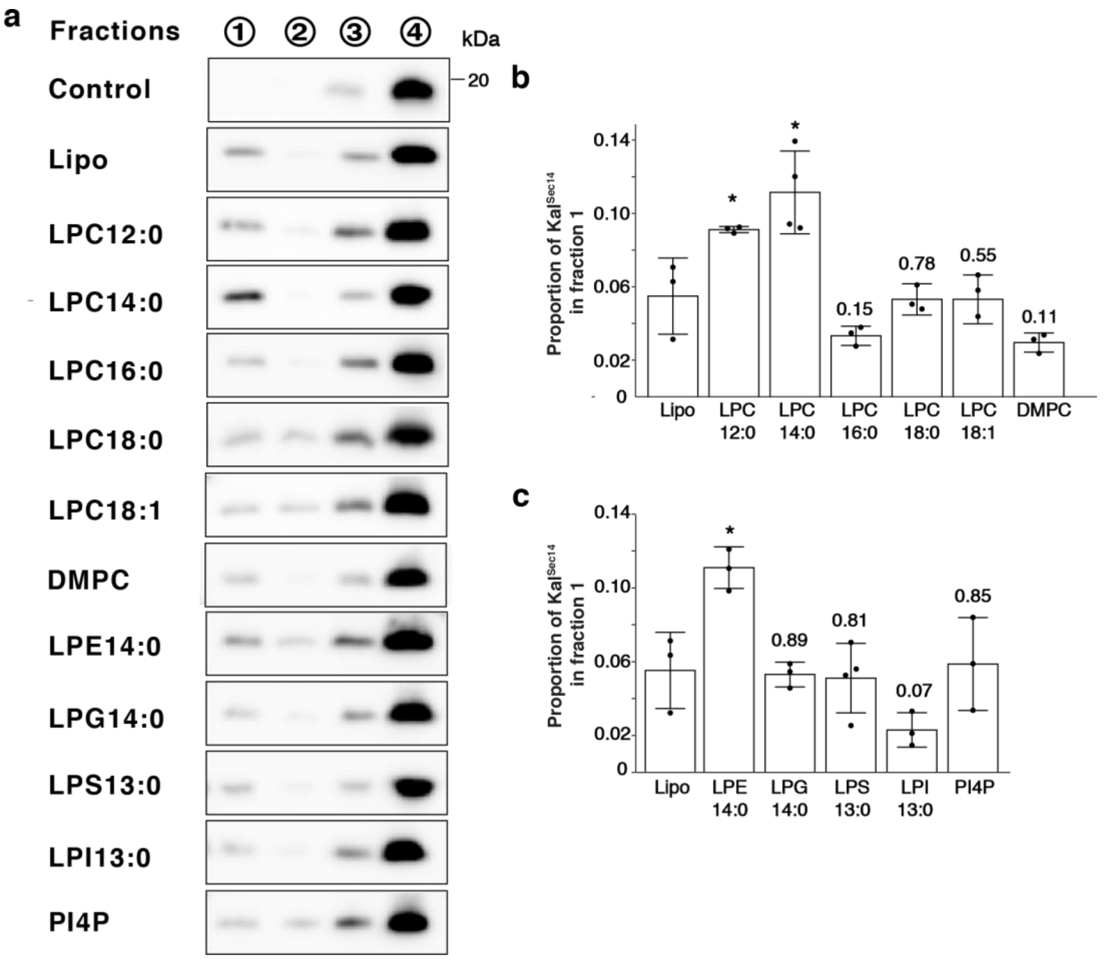

Supplementary Figure 9. Interaction of Kal^{Sec14} with phospholipids.

a. Representative Western blots from flotation assays with Kal^{Sec14} alone (Control), and Kal^{Sec14} with the control lipid mix (Lipo) or with the lipid mix containing the indicated additional lipid. After binding and separation of liposome bound Kal^{Sec14} from unbound Kal^{Sec14} on Accudenz gradients, the protein content of four gradient fractions was determined by immunoblot; liposomes were recovered from the top fraction (fraction 1). Source data are provided as a Source Data file.

b, c. Quantified group data showing protein content of the top fraction for each condition shown in panel *A*. Stated value is normalized to total protein recovered from each gradient. Data represent the mean ± S.D. of three (n = 3 for control, Lipo, LPC12:0, LPC16:0, LPC18:0, LPC18:1, DMPC, LPE14:0, LPG14:0, LPI13:0 and PI4P) or four (n = 4 for LPC14:0 and

LPS13:0) independent experiments. Black dots indicate individual data points. *P*-values were determined using two-tailed Student's *t* test. Statistical significance was determined *versus* Lipo mix as values of $p < 0.03$ (*$p < 0.03$; listed if considered not statistically significant).

a

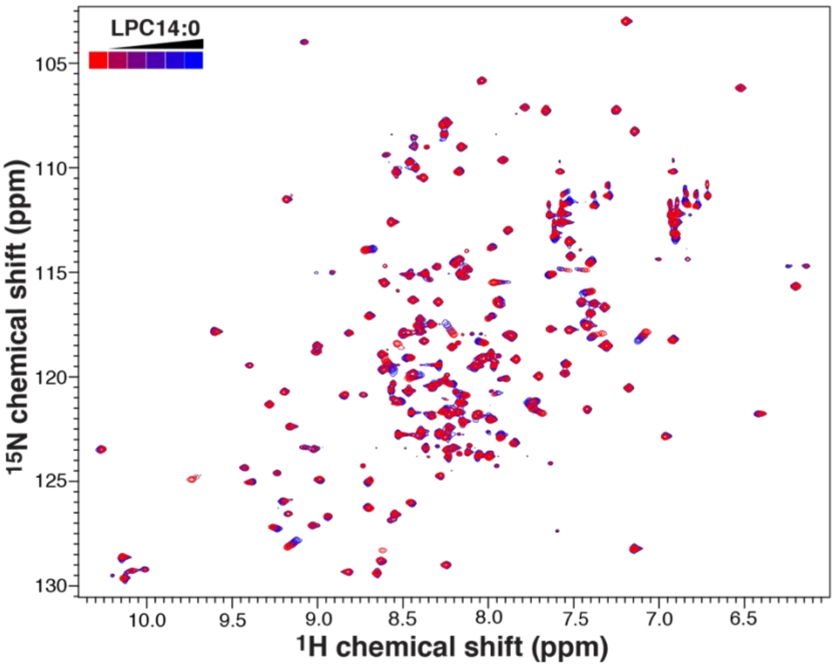
LPC14:0
105
110
115
120
125
130
15N chemical shift (ppm)
10.0 9.5 9.0 8.5 8.0 7.5 7.0 6.5
1H chemical shift (ppm)

b

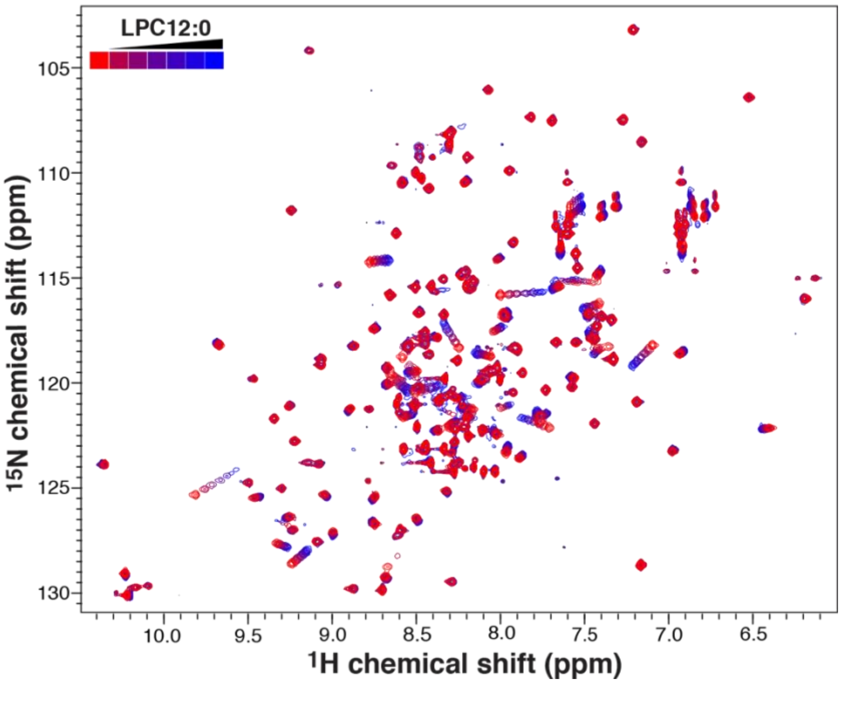
LPC12:0
105
110
115
120
125
130
15N chemical shift (ppm)
10.0 9.5 9.0 8.5 8.0 7.5 7.0 6.5
1H chemical shift (ppm)

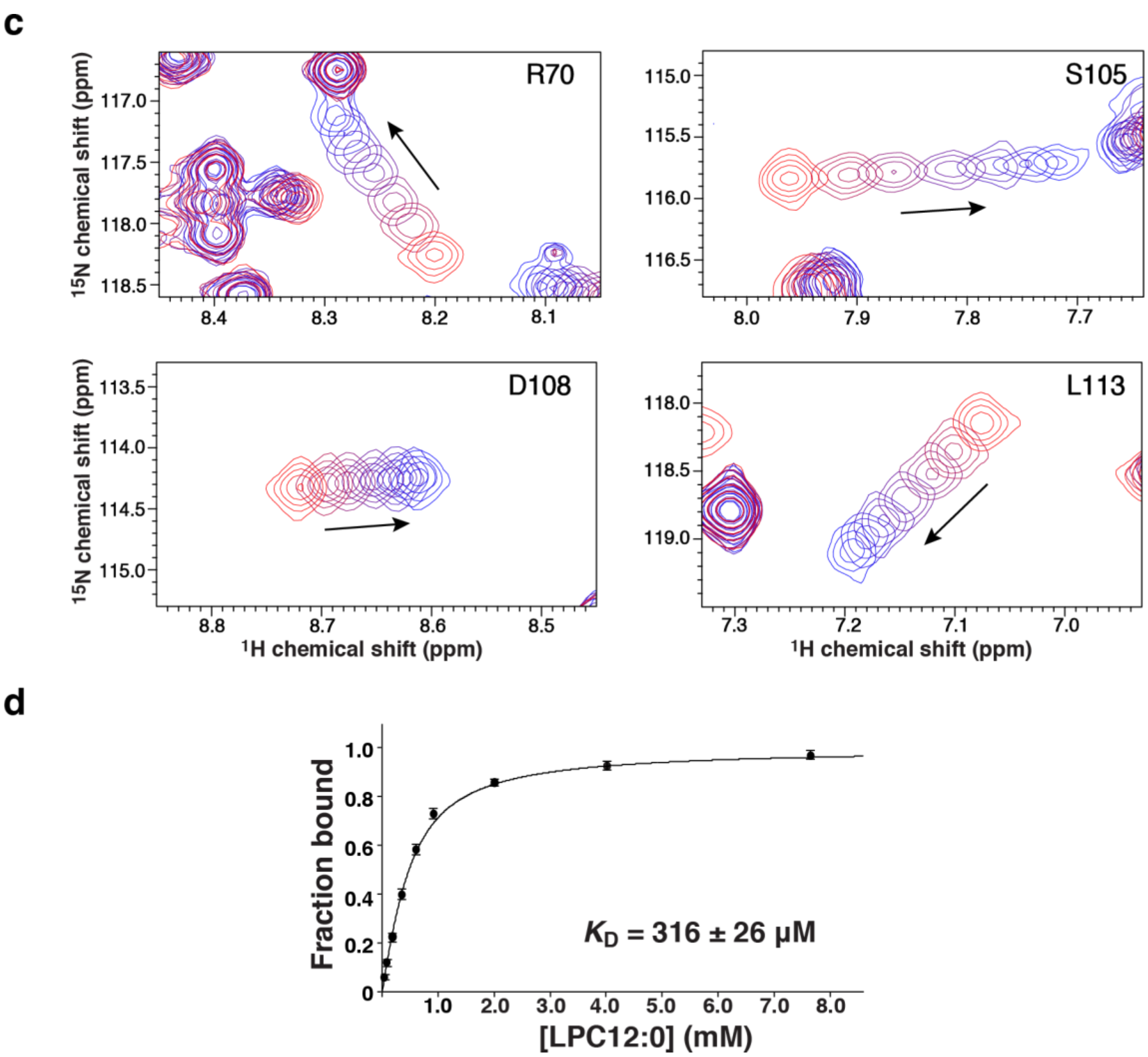

Supplementary Figure 10. Kal^{bSec14} binds LPC14:0 and LPC12:0.

a, b. Series of ^{1}H-^{15}N HSQC spectra recorded during titration of Kal^{bSec14} with increasing amounts of LPC14:0 (a) or LPC12:0 (b).

c. Four residues of Kal^{bSec14} that demonstrate CSPs when titrated with increasing amounts of LPC12:0 (1:20 final molar ratio). For each residue, the cross peaks are color-ramped from red to blue with increasing LPC12:0 concentrations, as indicated by arrow.

d. A plot of normalized global fitting of the averaged CSPs ($\Delta\delta_{obs}/\Delta\delta_{max}$) as a function of LPC12:0 concentration to estimate the K_D of binding. The fitting data and the K_D value represent the mean ± S.D. of the CSP data fitting for individual residues (n = 19). Source data are provided as a Source Data file).

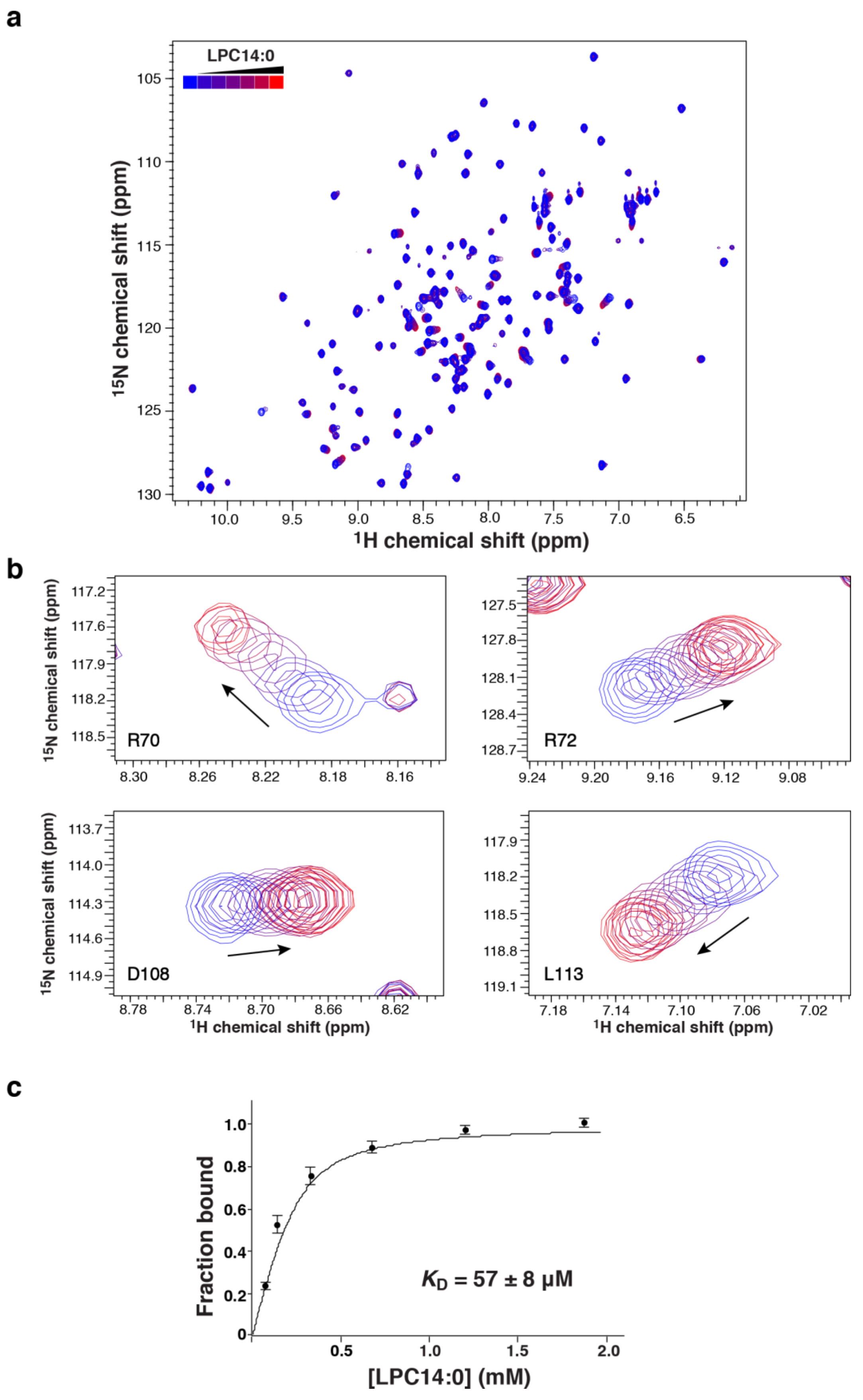
a
LPC14:0
105
110
115
120
125
130
15N chemical shift (ppm)
10.0
9.5
9.0
8.5
8.0
7.5
7.0
6.5
1H chemical shift (ppm)
b
R70
R72
D108
L113
15N chemical shift (ppm)
1H chemical shift (ppm)
c
Fraction bound
[LPC14:0] (mM)
K_D = 57 ± 8 μM

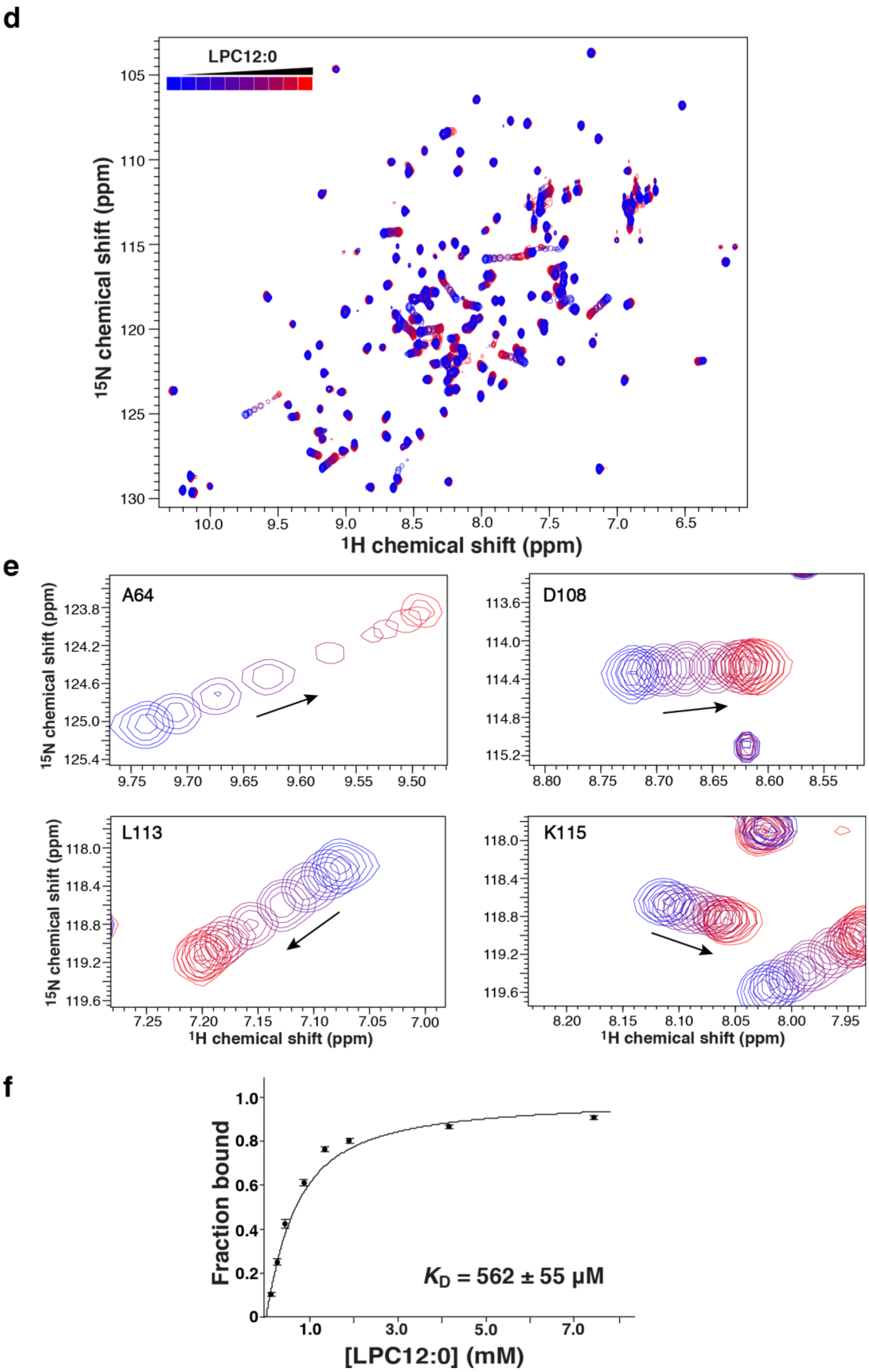

d
LPC12:0
15N chemical shift (ppm)
1H chemical shift (ppm)
e
A64
D108
L113
K115
15N chemical shift (ppm)
1H chemical shift (ppm)
f
Fraction bound
[LPC12:0] (mM)
K_D = 562 ± 55 μM

Supplementary Figure 11. Kal^{Sec14} binds LPC14:0 and LPC12:0.

a, d. Series of ^{1}H-^{15}N HSQC spectra recorded during titration of Kal^{Sec14} with increasing amounts of LPC14:0 (a) or LPC12:0 (d).

b, e. Four residues of Kal^{Sec14} that demonstrate CSPs when titrated with increasing amounts of LPC14:0 (b; 1:8 final molar ratio) or LPC12:0 (e; 1:21 final molar ratio). For each residue, the cross peaks are color-ramped from blue to red with increasing LPC14:0 or LPC12:0 concentrations, as indicated by arrow.

c, f. Plots of normalized global fitting of the averaged CSPs ($\Delta\delta_{obs}/\Delta\delta_{max}$) as a function of LPC14:0 (c) or LPC12:0 (f) concentration to estimate the K_D of binding. The fitting data and the K_D values represent the mean ± S.D. of the CSP data for individual residues (n = 5 for LPC14:0; n = 5 for LPC12:0). Source data are provided as a Source Data file.

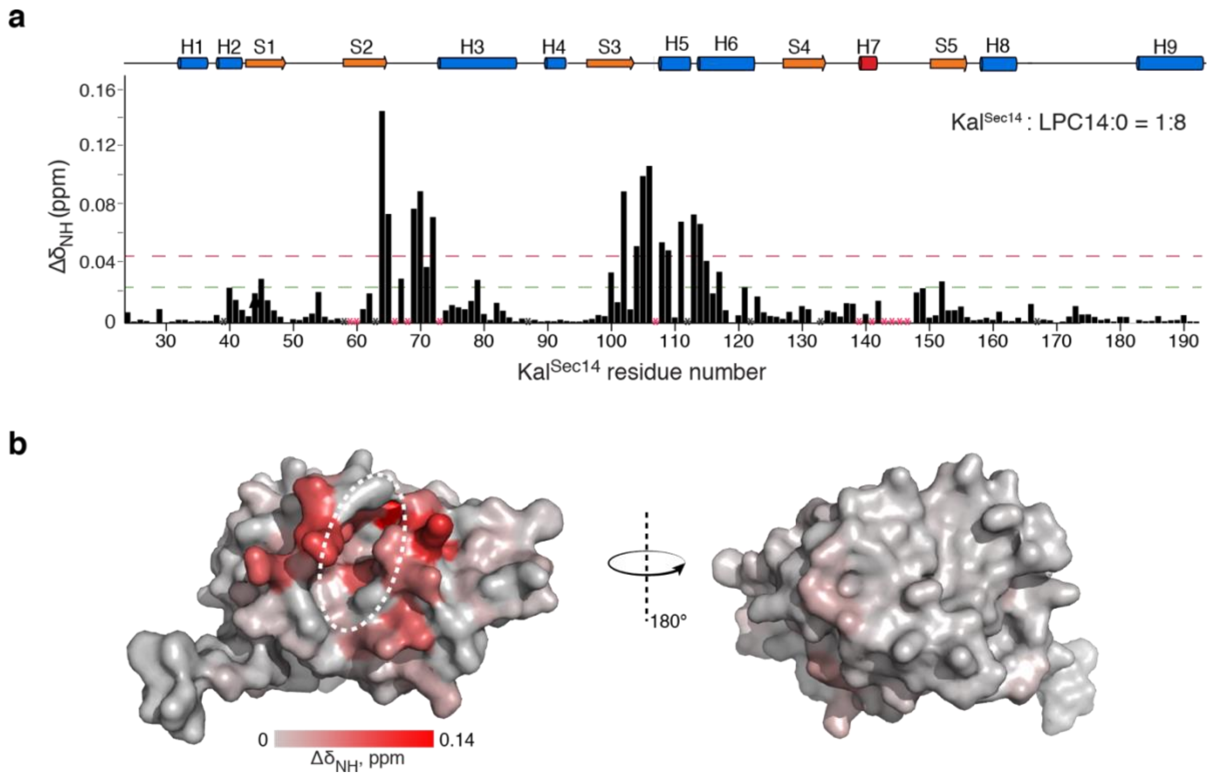

Supplementary Figure 12. Kal^{Sec14} binds LPC14:0 via the surface groove.

a. Plot of per-residue backbone CSPs between free and LPC14:0-bound states of Kal^{Sec14}. Proline residues and residues missing backbone assignment are indicated by asterisks (black, proline; red, unassigned). Dashed green and red lines indicate CSP values within one (1σ) and two (2σ) S.D. of the average CSP (0.022 ppm) among all assigned residues, respectively. Source data are provided as a Source Data file.

b. Surface representation of Kal^{Sec14} colored according to CSPs induced by LPC14:0 binding, from light gray (no observed CSP) to red (maximum CSP). Dashed ellipse indicates the surface groove.

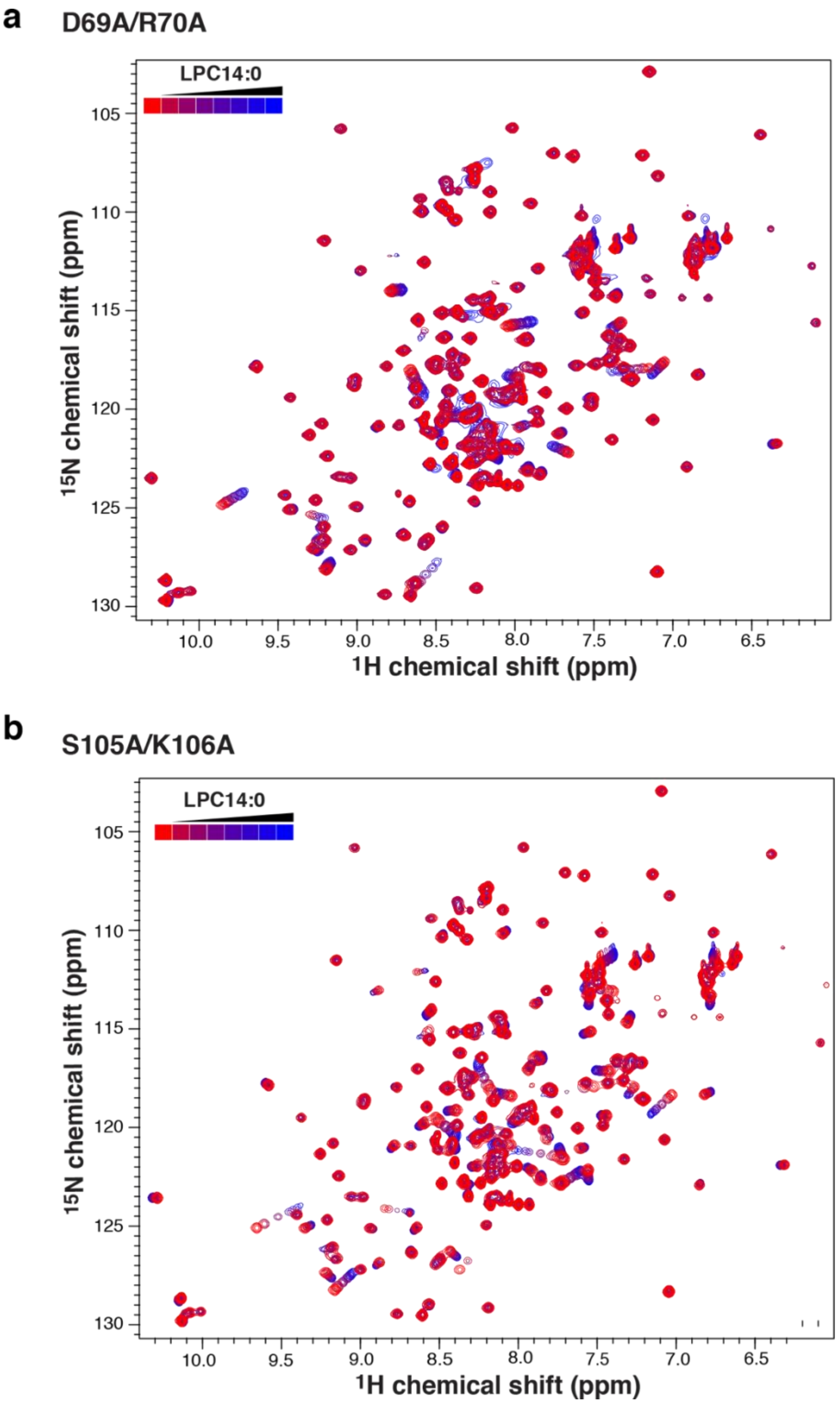

Supplementary Figure 13. NMR titration of LPC14:0 to the Kal^{bSec14} surface groove mutants.

Series of ^{1}H-^{15}N HSQC spectra recorded during titration of Kal^{bSec14}D69A/R70A (a) and Kal^{bSec14}S105A/K106A (b) with increasing amounts of LPC14:0.

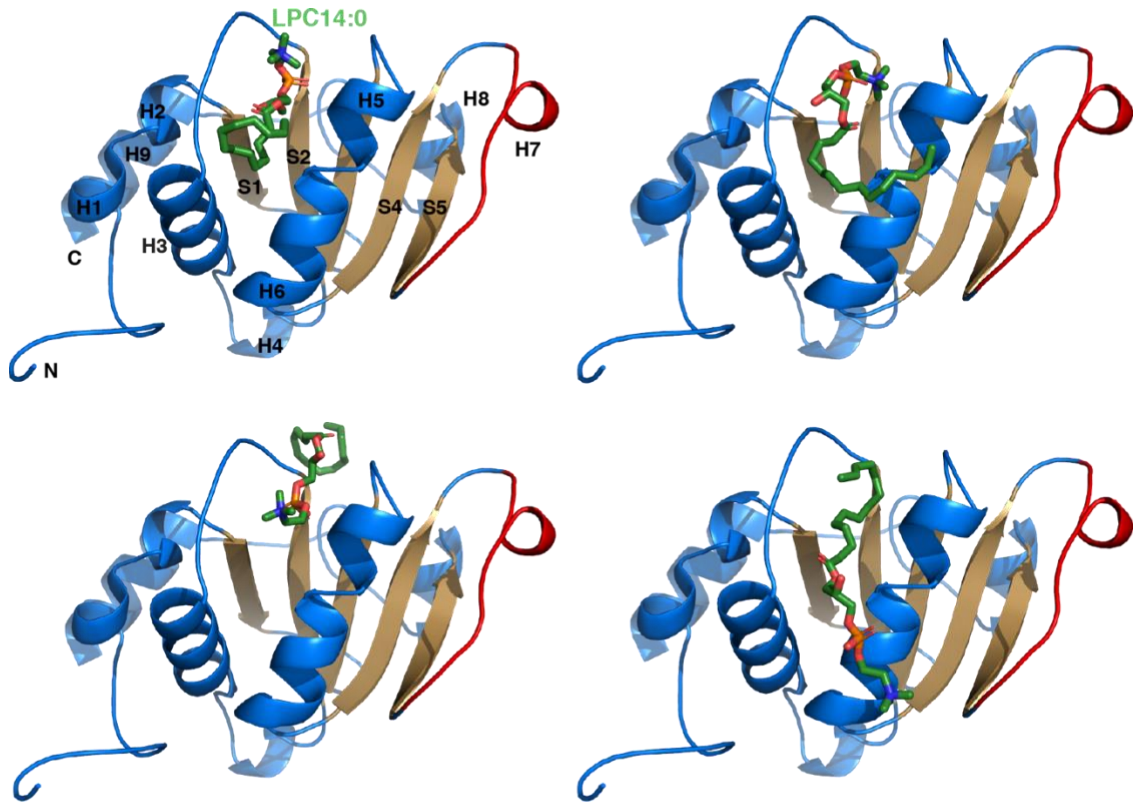

Supplementary Figure 14. Molecular docking of LPC14:0 onto the structure of Kal^{bSec14}.

Four top-ranked docked poses of LPC14:0 determined by AutoDock Vina [9] are shown as licorice sticks in the surface cavity of Kal^{bSec14}.

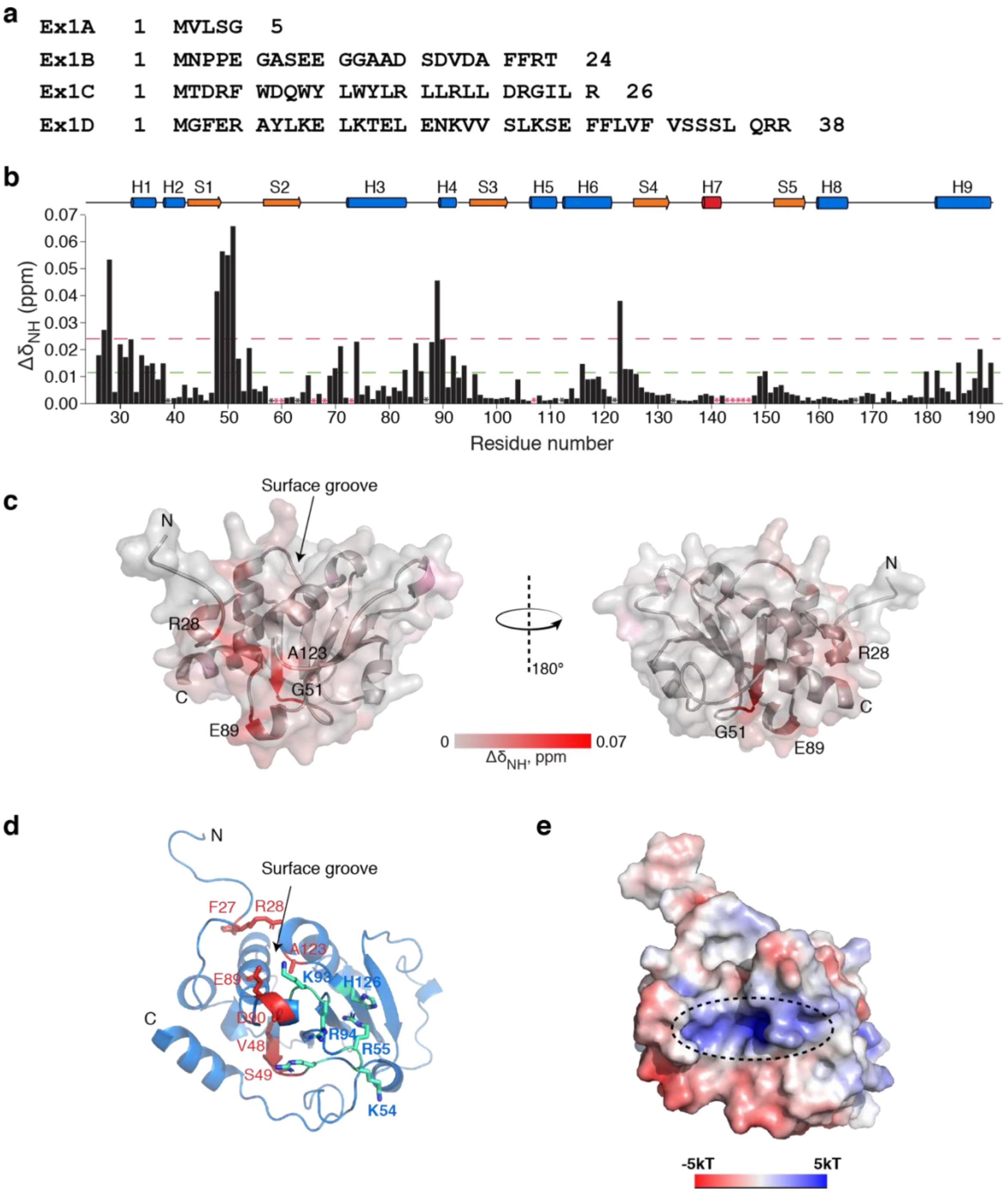

Supplementary Figure 15. Effect of Ex1B front peptide on the LPC binding.

a. Sequences of the four front peptides encoded by the rat *KALRN* sequence. The human orthologs of rat Ex1B and rat Ex1C encode identical peptides. The Ex1B peptide is negatively charged at physiologic pH (isoelectric point of 3.5), while the Ex1C peptide is positively charged (isoelectric point of 10.1).

b. Plot of per-residue backbone CSPs comparing Kal^{Sec14} and Kal^{bSec14}. Proline residues and residues missing backbone assignment are indicated by asterisks (black, proline; red, unassigned). Dashed green and red lines indicate CSP values within one (1σ) and two (2σ) S.D. of the average CSP (0.012 ppm) among all assigned residues, respectively. The observed chemical shift differences between the spectra of Kal^{bSec14} and Kal^{Sec14} do not appear to be caused by a global conformational change in the protein, enabling faithful transfer of the assignments from Kal^{Sec14} to Kal^{bSec14}. Source data are provided as a Source Data file.

c. Surface representation of Kal^{bSec14} colored according to CSPs presented in panel b, from light gray (no observed CSP) to red (maximum CSP).

d. Residues exhibiting the largest CSPs (>2σ) are shown as red licorice sticks. They are located in four regions, the assigned N-terminal loop (F27 and R28), strand S1 and the following S1–S2 loop (V48–G51), helix H4 (E89 and D90) and the H6–S4 loop (A123). The basic residues located adjacent to these CSP-sensitive regions are shown as green licorice sticks.

e. Molecular surface representation of Kal^{bSec14} shown in an orientation similar to that in *d* and colored according to the local electrostatic potential calculated with program ABPS[10]. Dashed ellipse indicates the positively charged patch in panel d.

a

Name	Uniprot ID	Isoform	Front Peptide Residues	Sequence Identity to Kal^{bSec14} (%)	RMSD of C_α atoms to Kal^{bSec14} (Å)
TRIO	O75962-1	1	61	76.7	1.4
MCF2L2	Q86YR7-1	1	36	36.1	1.3
MCF2	P10911-3	3	–	37.4	1.7
MCF2L	O15068-1	1	67	34.6	1.5
SESD1	Q86VW0-1	1	–	44.5	1.7

b

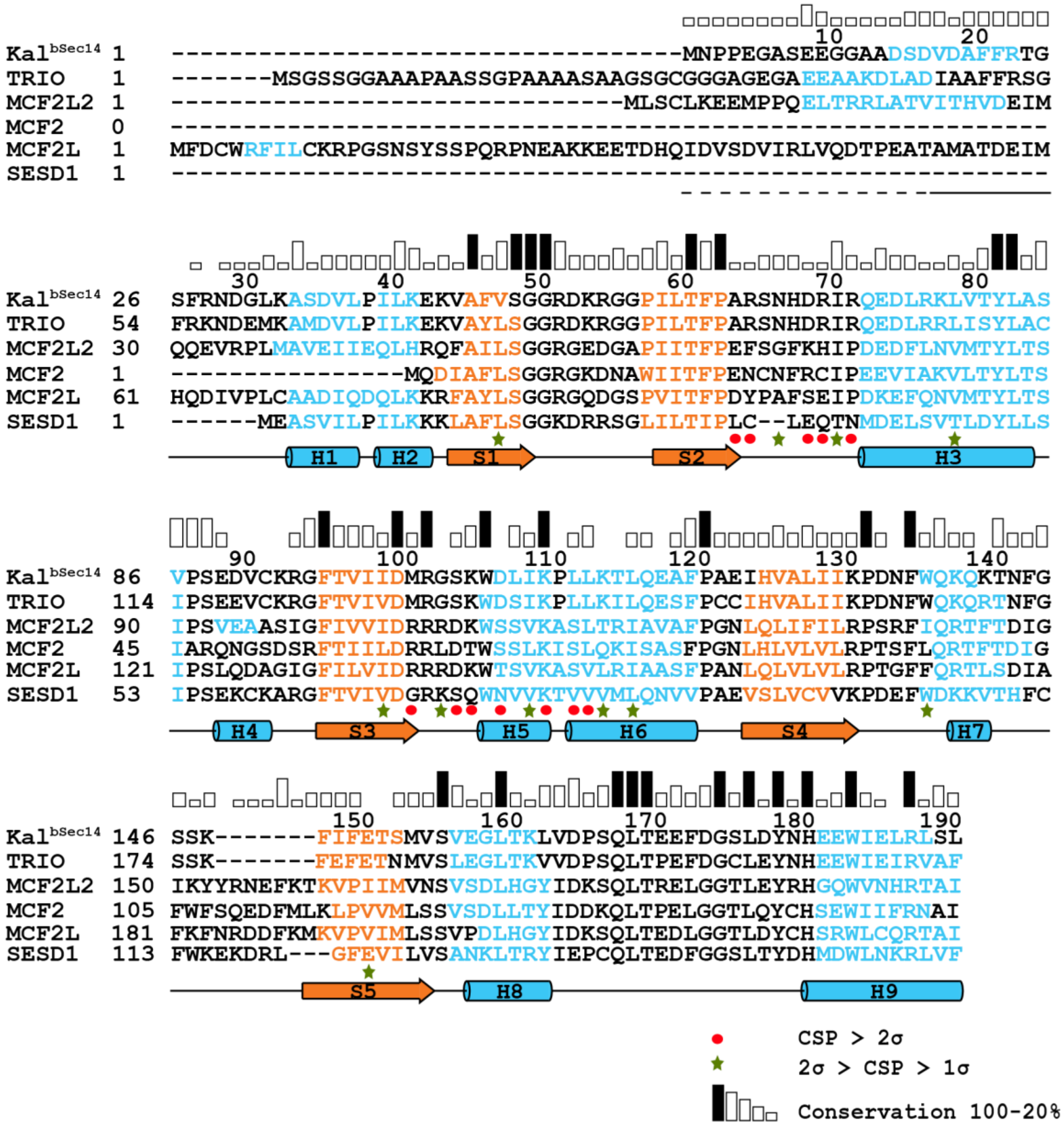

c

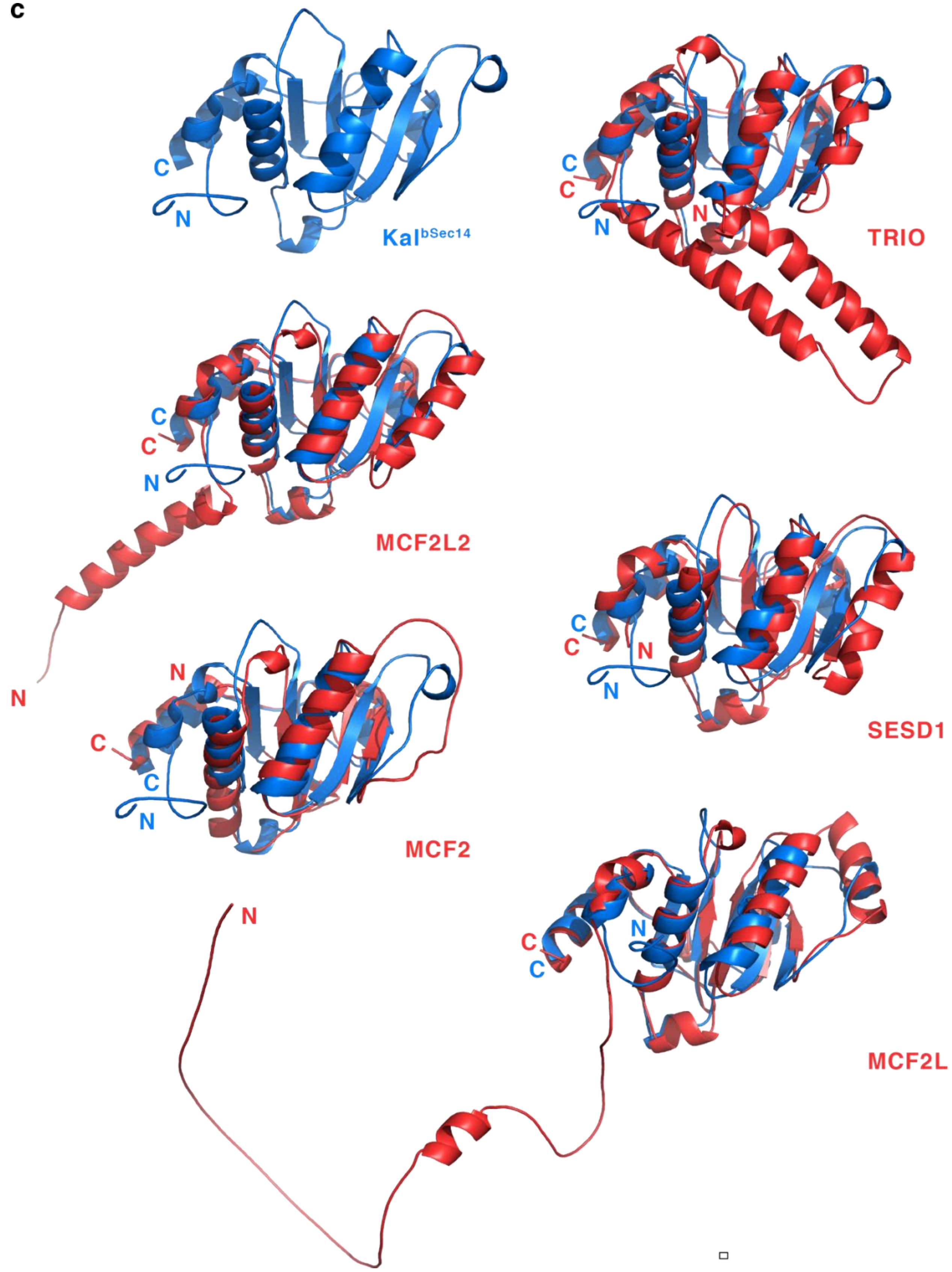

Supplementary Figure 16. Sequence and structural conservation of Kal^{bSec14} and other mammalian SecSR proteins.

a. Sequence identity of the putative SecSR domains in five human SecSR-containing proteins to Kal^{bSec14}. Three of them possess a front peptide.

b. Sequence and structural conservation among Kal^{bSec14} and the putative Sec14/SR domains in these SecSR proteins. Sequence conservation is shown as a bar graph, with black bars indicating identity among those proteins. Secondary-structure assignments of Kal^{bSec14} from the crystal structure are shown as blue cylinders (helices), orange arrows (β strands) and broken lines (disordered regions). Predicted secondary-structure elements were obtained using AlphaFold [11] and are indicated by color (blue: helix; orange: strand). The Kal^{bSec14} residues with large LPC14:0-induced CSPs are indicated by red dots (CSP > 2σ) and green asterisks (2σ > CSP > 1σ).

c. Superposition of the structure of Kal^{bSec14} and AlphaFold-predicted structures of other SecSR proteins. Overall rmsd of C_α atoms of the SecSR proteins to Kal^{bSec14} are listed in panel a.

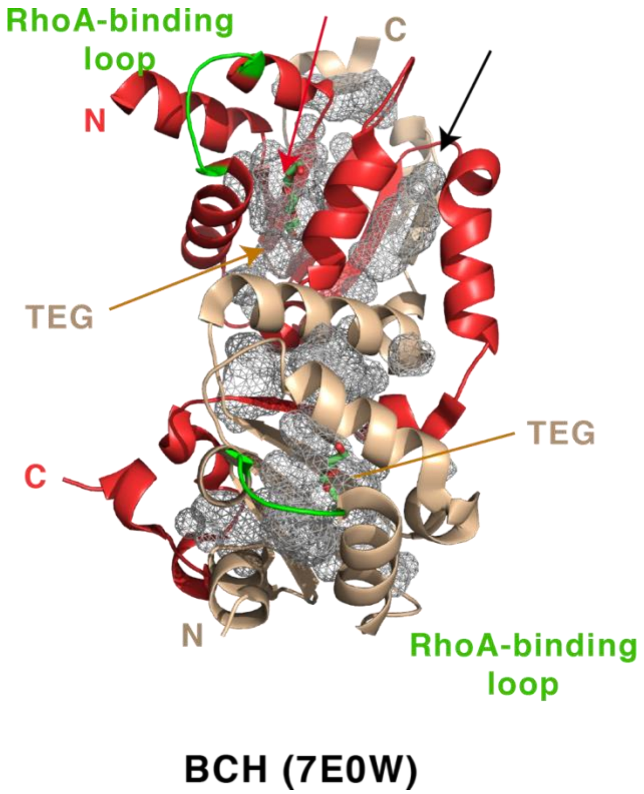

Supplementary Figure 17. Pockets in the structure of the BCH domain of p50RhoGAP.

Structure of the BCH domain of p50RhoGAP (PDB ID code 7E0W; rmsd of 2.8 Å over 151 C_{α} atoms with that of Kal^{bSec14}) with its inner and surface cavities shown in a surface representation calculated by Pymol. The two monomers of the BCH domain are colored in red and grey, respectively. The locations of the presumed surface groove and the canonical ligand binding site are indicated by red and black arrows, respectively. The TEG molecules co-purified with the protein are found in a pocket similar to the Kal^{bSec14} surface groove and shown as licorice sticks. The RhoA-binding loops are colored in green.

Supplementary Table 1. Summary of crystallographic analysis.

	Native	SeMet-SAD (Peak)
**Data collection**		
Wavelength (Å)	1.19499	0.97915
Space group	*P1*	*P1*
Cell dimensions (Å)		
a, *b*, *c* (Å)	72.16, 82.42, 83.27	72.20, 81.79, 83.73
α, β, γ (°)	81.09, 71.79, 79.88	82.21, 70.85, 80.26
Resolution (Å)	78.64–1.89 (1.92–1.89)	80.32-2.22 (2.26–2.22)
R_{sym} (%)	6.1 (129.4)	19.0 (237.2)
Mean ($I/\sigma I$)	31.6 (2.2)	22.3 (2.1)
$CC_{1/2}$	1.000 (0.907)	0.999 (0.815)
Completeness (%)	95.9 (94.0)	98.0 (97.4)
Multiplicity	18.3 (19.0)	38.8 (41.1)
Anomalous completeness (%)		97.5 (97.0)
Anomalous multiplicity		20.0 (20.6)
CC_{ano}		0.774 (0.345)
**Refinement**		
Resolution (Å)	61.01–1.89	
No. reflections (|F|>0σ)	137,668	
R_{work}/R_{free} (%)	19.5/21.8	
No. atoms		
Protein	10,703	
Water	1,440	
Average B-factors (Å^2)		
Protein	47.81	
Water	53.71	
Wilson B-factors (Å^2)	36.31	
R.m.s. deviations		
Bond lengths (Å)	0.010	
Bond angles (°)	0.99	
Ramachandran plot (%)		
Favored (%)	99.08	
Allowed (%)	100.0	
Outliers (%)	0.00	

Values in parentheses are for the highest-resolution shell. SAD, single-wavelength anomalous dispersion.

Supplementary Table 2. List of the detergents and the lipids used in this work.

Name	Full name	Company/ Catalog number	Chemical structure*
FC10	Decylphosphocholine	Cube Biotech #16041	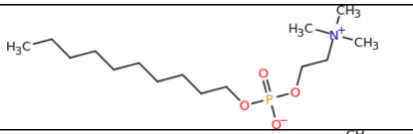
FC12	Dodecylphosphocholine	Cube Biotech #16041	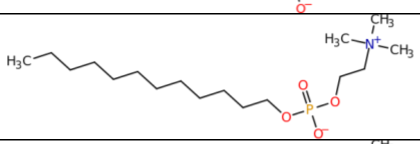
FC14	Tetradecylphosphocholine	Cube Biotech #16209	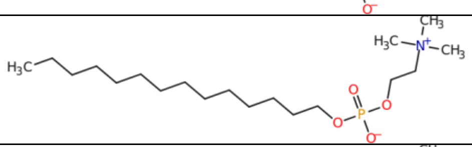
FC16	Hexadecylphosphocholine	Cube Biotech #16307	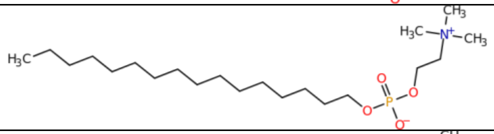
LPC12:0	1-lauroyl-2-hydroxy-sn-glycero-3-phosphocholine	Avanti Polar Lipid #855475	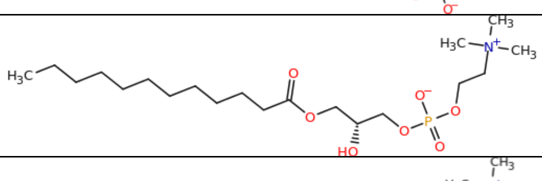
LPC14:0	1-myristoyl-2-hydroxy-sn-glycero-3-phosphocholine	Avanti Polar Lipid #855575	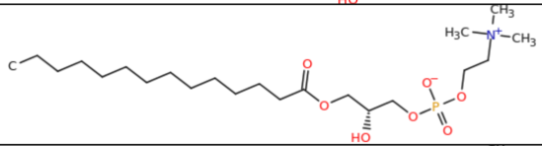
LPC16:0	1-palmitoyl-2-hydroxy-sn-glycero-3-phosphocholine	Avanti Polar Lipid #855675	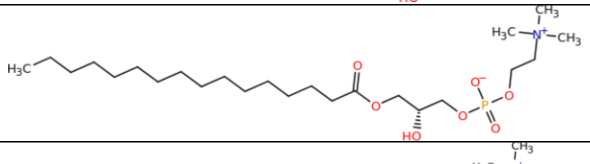
LPC18:0	1-stearoyl-2-hydroxy-sn-glycero-3-phosphocholine	Avanti Polar Lipid #855675	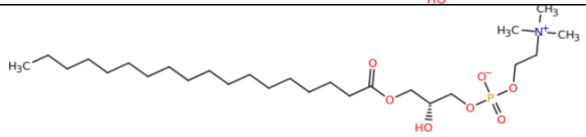
LPC18:1	1-oleoyl-2-hydroxy-sn-glycero-3-phosphocholine	Avanti Polar Lipid #845875	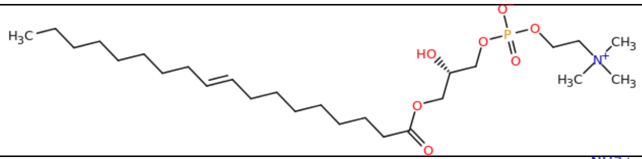
LPE14:0	1-myristoyl-2-hydroxy-sn-glycero-3-phosphoethanolamine	Avanti Polar Lipid #856735	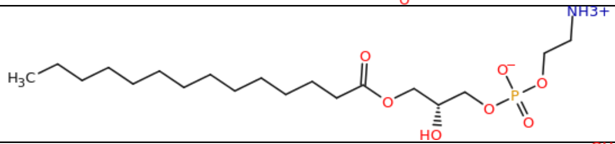
LPG14:0	1-myristoyl-2-hydroxy-sn-glycero-3-phospho-(1'-rac-glycerol)	Avanti Polar Lipid #858120	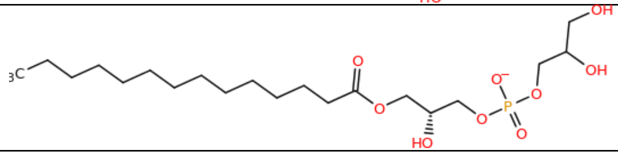

LPS13:0	1-tridecanoyl-sn-glycero-3-phospho-L-serine	Avanti Polar Lipid #858140	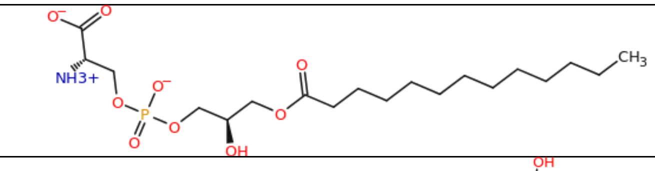
LPI13:0	1-tridecanoyl-2-hydroxy-sn-glycero-3-phospho-(1'-myo-inositol)	Avanti Polar Lipid # 850101	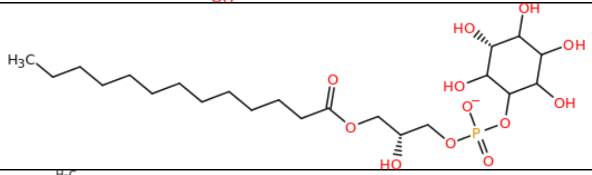
DMPC	1,2-dimyristoyl-sn-glycero-3-phosphocholine	Avanti Polar Lipid # 850345	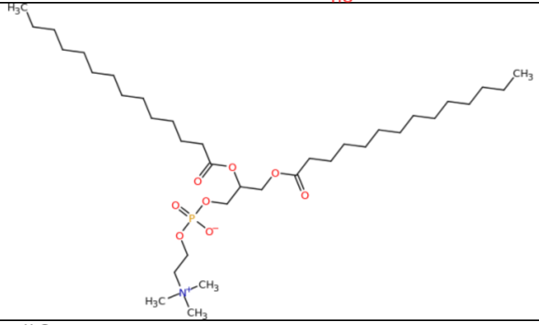
PI4P	L-α-phosphatidylinositol-4-phosphate	Avanti Polar Lipid # 840045	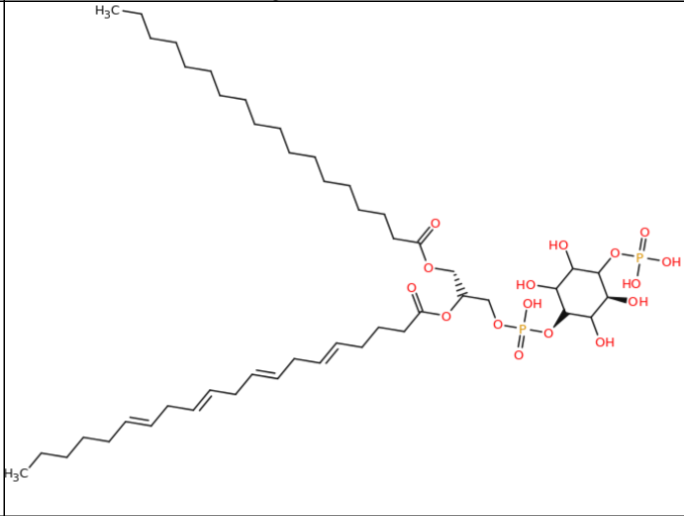
DOPC	1,2-dioleoyl-sn-glycero-3-phosphocholine	Avanti Polar Lipid # 850375	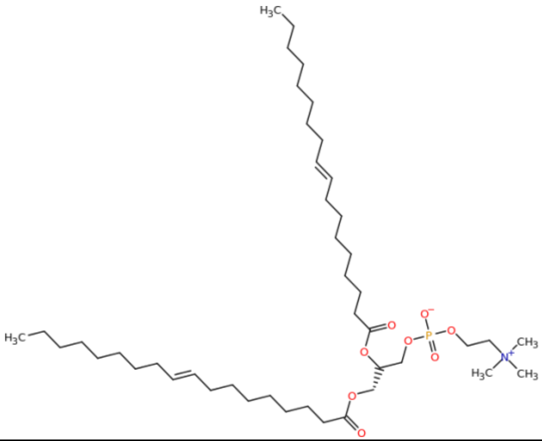

DOPE	1,2-dioleoyl-sn-glycero-3-phosphoethanolamine	Avanti Polar Lipid # 850725	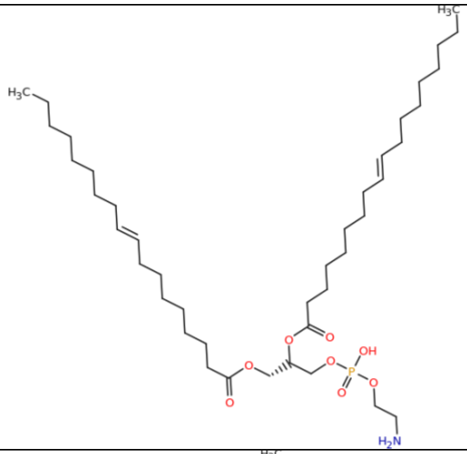
DOPS	1,2-dioleoyl-sn-glycero-3-phospho-L-serine	Avanti Polar Lipid # 840035	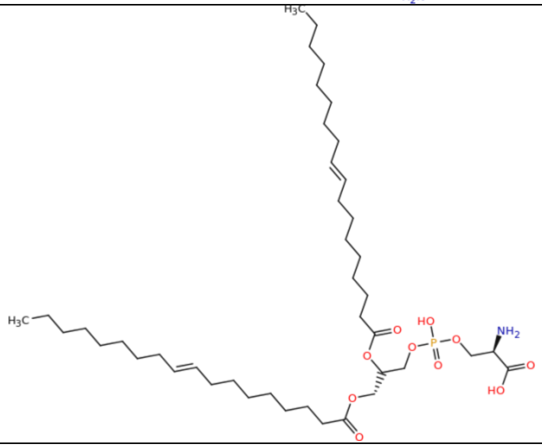
Cholesterol	Cholesterol (ovine)	Avanti Polar Lipid # 700000	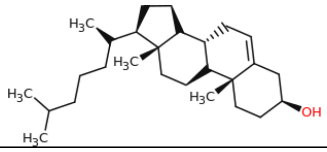
Liss Rhod PE	1,2-dioleoyl-sn-glycero-3-phosphoethanolamine-N-(lissamine rhodamine B sulfonyl) (ammonium salt)	Avanti Polar Lipid # 810150	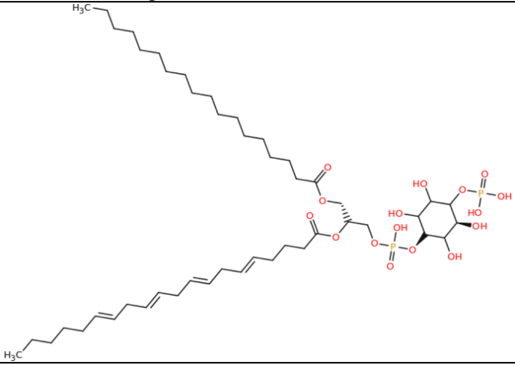

Supplementary References

1. Ho, B.K. & Gruswitz, F. HOLLOW: generating accurate representations of channel and interior surfaces in molecular structures. *BMC Struct. Biol.* **8**, 49 (2008).
2. Dundas, J. et al. CASTp: computed atlas of surface topography of proteins with structural and topographical mapping of functionally annotated residues. *Nucleic Acids Res.* **34**, W116-118 (2006).
3. Sha, B., Phillips, S.E., Bankaitis, V.A. & Luo, M. Crystal structure of the *Saccharomyces cerevisiae* phosphatidylinositol-transfer protein. *Nature* **391**, 506-510 (1998).
4. He, X., Lobsiger, J. & Stocker, A. Bothnia dystrophy is caused by domino-like rearrangements in cellular retinaldehyde-binding protein mutant R234W. *Proc. Natl. Acad. Sci. USA* **106**, 18545-18550 (2009).
5. Schaaf, G. et al. Functional anatomy of phospholipid binding and regulation of phosphoinositide homeostasis by proteins of the sec14 superfamily. *Mol. Cell* **29**, 191-206 (2008).
6. Khan, D. et al. A Sec14-like phosphatidylinositol transfer protein paralog defines a novel class of heme-binding proteins. *Elife* **9,** e57081 (2020).
7. D'Angelo, I., Welti, S., Bonneau, F. & Scheffzek, K. A novel bipartite phospholipid-binding module in the neurofibromatosis type 1 protein. *EMBO Rep.* **7**, 174-179 (2006).
8. Kono, N. et al. Impaired alpha-TTP-PIPs interaction underlies familial vitamin E deficiency. *Science* **340**, 1106-1110 (2013).
9. Trott, O. & Olson, A.J. AutoDock Vina: improving the speed and accuracy of docking with a new scoring function, efficient optimization, and multithreading. *J. Comput. Chem.* **31**, 455-461 (2010).
10. Baker, N.A., Sept, D., Joseph, S., Holst, M.J. & McCammon, J.A. Electrostatics of nanosystems: application to microtubules and the ribosome. *Proc. Natl. Acad. Sci. USA* **98**, 10037-10041 (2001).
11. Jumper, J. et al. Highly accurate protein structure prediction with AlphaFold. *Nature* **596**, 583-589 (2021).
