# Supplementary material for: Structure of the Sec14 domain of Kalirin reveals a distinct class of lipid-binding module in RhoGEFs
Source: Nat Commun. 2023 Jan 6;14:96. doi: 10.1038/s41467-022-35678-4 (PMC9823006; doi:10.1038/s41467-022-35678-4)
Supplement: Supplementary file 3 — Reporting Summary [file 41467_2022_35678_MOESM3_ESM.pdf]

nature portfolio

Corresponding author(s): Bing Hao

Last updated by author(s): Nov 28, 2022

Reporting Summary

Nature Portfolio wishes to improve the reproducibility of the work that we publish. This form provides structure for consistency and transparency in reporting. For further information on Nature Portfolio policies, see our Editorial Policies and the Editorial Policy Checklist.

Statistics

For all statistical analyses, confirm that the following items are present in the figure legend, table legend, main text, or Methods section.

n/a	Confirmed	
☐	☒	The exact sample size (*n*) for each experimental group/condition, given as a discrete number and unit of measurement
☐	☒	A statement on whether measurements were taken from distinct samples or whether the same sample was measured repeatedly
☐	☒	The statistical test(s) used AND whether they are one- or two-sided<br>*Only common tests should be described solely by name; describe more complex techniques in the Methods section.*
☒	☐	A description of all covariates tested
☒	☐	A description of any assumptions or corrections, such as tests of normality and adjustment for multiple comparisons
☐	☒	A full description of the statistical parameters including central tendency (e.g. means) or other basic estimates (e.g. regression coefficient) AND variation (e.g. standard deviation) or associated estimates of uncertainty (e.g. confidence intervals)
☐	☒	For null hypothesis testing, the test statistic (e.g. *F*, *t*, *r*) with confidence intervals, effect sizes, degrees of freedom and *P* value noted<br>*Give P values as exact values whenever suitable.*
☒	☐	For Bayesian analysis, information on the choice of priors and Markov chain Monte Carlo settings
☒	☐	For hierarchical and complex designs, identification of the appropriate level for tests and full reporting of outcomes
☒	☐	Estimates of effect sizes (e.g. Cohen's *d*, Pearson's *r*), indicating how they were calculated

Our web collection on statistics for biologists contains articles on many of the points above.

Software and code

Policy information about availability of computer code

Data collection	LSDC for X-ray data collection . VnmrJ and TopSpin for NMR data collection.
Data analysis	autoPROC (version 1.0.5) and Fast DP (version 1.6.1) for X-ray data processing. PHENIX (version 1.19.2-4158), CCP4 (version 7.0.078), BUSTER (version 2.10.3) and COOT (version 0.8.9.2 EL) for structure determination, refinement and model building. PyMol (version 2.3.0) for making structural figures. NMRPipe (version 11.1), CARA (version 1.9.1.7) and CcpNmr (version 2.5.2) for NMR data processing and analyses. AutoDock Vina (version 1.1.2) for molecular docking. GeneSys (version 4.3.10.0) for Western blot analyses.

For manuscripts utilizing custom algorithms or software that are central to the research but not yet described in published literature, software must be made available to editors and reviewers. We strongly encourage code deposition in a community repository (e.g. GitHub). See the Nature Portfolio guidelines for submitting code & software for further information.

Data

Policy information about availability of data

All manuscripts must include a data availability statement. This statement should provide the following information, where applicable:

- Accession codes, unique identifiers, or web links for publicly available datasets
- A description of any restrictions on data availability
- For clinical datasets or third party data, please ensure that the statement adheres to our policy

The atomic coordinates and structure factors of KalbSec14 have been deposited in the RCSB Protein Data Bank under accession code 7UR2 [https://doi.org/10.2210/pdb7UR2/pdb] (The Sec14 domain of Kalirin). The backbone 1H, 13C and 15N resonance assignments of KalSec14 have been deposited to BMRB

under the accession number 51382 [https://bmrb.io/data_library/summary/index.php?bmrbId=51382] (The NMR backbone assignments of the Kalirin Sec14 domain). Atomic coordinates for the previously determined Sec14 domain containing proteins were accessed from Protein Data Bank under accession codes 1AUA [https://doi.org/10.2210/pdb1AUA/pdb], 1OIZ [https://doi.org/10.2210/pdb1OIZ/pdb], 1O6U [https://doi.org/10.2210/pdb1O6U/pdb], 2D4Q [https://doi.org/10.2210/pdb1D4Q/pdb], 3B7N [https://doi.org/10.2210/pdb3B7N/pdb], 3HY5 [https://doi.org/10.2210/pdb3HY5/pdb], 3W67 [https://doi.org/10.2210/pdb3W67/pdb], 6W32 [https://doi.org/10.2210/pdb6W32/pdb], and 7E0W [https://doi.org/10.2210/pdb7E0W /pdb].

Human research participants

Policy information about studies involving human research participants and Sex and Gender in Research.

Reporting on sex and gender	Not applicable.
Population characteristics	Not applicable.
Recruitment	Not applicable.
Ethics oversight	Not applicable.

Note that full information on the approval of the study protocol must also be provided in the manuscript.

Field-specific reporting

Please select the one below that is the best fit for your research. If you are not sure, read the appropriate sections before making your selection.

☒ Life sciences ☐ Behavioural & social sciences ☐ Ecological, evolutionary & environmental sciences

For a reference copy of the document with all sections, see nature.com/documents/nr-reporting-summary-flat.pdf

Life sciences study design

All studies must disclose on these points even when the disclosure is negative.

Sample size	No statistical method was used to predetermine the sample size. Standard considerations based on expected variations from previous experiments (10.1074/jbc.M115.636746) were applied to determine the necessary repeats to ensure reproducibility and statistical significance. The corresponding number of biological replicates that was analyzed is indicated in the Figure legends.
Data exclusions	All X-ray and NMR data were processed using standard protocols and no data were excluded intentionally unless extreme outliers detected by the softwares. No western blot data were excluded from analyses.
Replication	We have collected more than 40 native X-ray diffraction data sets and more than 50 heavy-atom and SeMet data sets. For Western blot experiments, we have indicated the number of independent biological replicates performed in the figure legends and the Source Data file. All replicates were performed using independently generated liposome mixtures. All attempts at replication were successful.
Randomization	For structure refinement, a random subset of 5% reflections was set aside by the program CCP4 for calculating Rfree value that are used to evaluate the quality of the final structural model. This process is not controlled and does not affect the final model calculation. No randomization was performed in other experiments.
Blinding	Blinding was not feasible for X-ray crystallography as we do not know which lipids will or will not bind, the same for the NMR titration experiments. Blinding was not relevant to other experiments in this study.

Reporting for specific materials, systems and methods

We require information from authors about some types of materials, experimental systems and methods used in many studies. Here, indicate whether each material, system or method listed is relevant to your study. If you are not sure if a list item applies to your research, read the appropriate section before selecting a response.

Materials & experimental systems

n/a	Involved in the study	
☐	☒	Antibodies
☒	☐	Eukaryotic cell lines
☒	☐	Palaeontology and archaeology
☒	☐	Animals and other organisms
☒	☐	Clinical data
☒	☐	Dual use research of concern

Methods

n/a	Involved in the study	
☒	☐	ChIP-seq
☒	☐	Flow cytometry
☒	☐	MRI-based neuroimaging

Antibodies

Antibodies used

Primary antibody to the Sec14 domain of Kalirin (so called CT302) was produced by immunizing rabbits (Covance Research Products, Denver, PA, USA) with purified rat KalSec14 (NHDR...RLSL; pGEX4T2-KalSec14P); after ammonium sulfate precipitation, affinity purification was carried out using rat Sec14 linked to Affi-Gel15 beads. Affinity-purified CT302 detects Kal7, Kal9, and Kal12. CT302 is registered in the Antibody Registry (RRID: AB_2801573) [https://antibodyregistry.org/search?q=kalirin&page=5] and was first described in the publication 10.1093/cercor/bhu182. Secondary HRP-tagged antibody used in this study is a goat anti-Rabbit IgG (H+L) (ThermoFisher Scientific, #31460).

Validation

CT302 were validated against recombinant proteins as well as Kalirin knockout mouse tissue. The purchased secondary antibody has been validated in multiple previous studies listed at the manufacturer's website (https://www.thermofisher.com/antibody/product/Goat-anti-Rabbit-IgG-H-L-Secondary-Antibody-Polyclonal/31460).
